# The Association of Growth and Maturation with Injury in Academy Soccer Players: A Narrative Review

**DOI:** 10.1007/s40279-025-02340-0

**Published:** 2025-11-14

**Authors:** Elliott C. R. Hall, Robert M. Erskine

**Affiliations:** 1 Faculty of Health Sciences and Sport, University of Stirling, Stirling, UK; 2 School of Sport and Exercise Sciences, Liverpool John Moores University, Liverpool, UK; 3 Institute of Sport, Exercise and Health, University College London, London, UK

## Abstract

**Background:**

The point of fastest growth during somatic maturation is termed ‘peak height velocity’ (PHV), and the chronological age at which this occurs varies considerably. Academy football (soccer) players are typically categorised by chronological age, yet many children of the same age will naturally mature and grow at different ages and rates, respectively, which could affect injury risk. However, despite nearly two decades of studies investigating the association of growth and maturation with injury in academy footballers, confusion remains.

**Objective:**

To critically appraise the literature concerning the association of maturity timing, maturity status and growth rate with injury in academy football.

**Methods:**

Scopus, PubMed, SPORTDiscus and CINAHL databases were screened from inception until April 2025. Study design, setting, sample size, methods for estimating maturity timing/maturity status/growth rate, and injury reporting were evaluated in this narrative review to determine individual study quality.

**Results:**

A total of 472 articles were screened with data extracted from 26 eligible studies published between 2007 and 2025.

**Conclusions:**

We found that (i) early maturing players suffer more soft-tissue injuries than on-time or late maturing players; (ii) more growth-related injuries occur circa-PHV than pre- or post-PHV, but post-PHV players generally have greater injury risk than circa- or pre-PHV; and (iii) fast growth (stature increase ≥ 7.2 cm per year) increases injury risk in academy footballers. However, all eligible studies demonstrated inherent limitations and none investigated the impact of maturity timing, maturity status or growth rate on injury in female academy players, indicating future research should address these issues.

**Supplementary Information:**

The online version contains supplementary material available at 10.1007/s40279-025-02340-0.

## Key Points

**Table:** 

Early maturing academy football players are more likely to suffer soft-tissue (muscle, ligament or tendon) injuries than their peers, who mature on time or late.
Whilst growth-related injuries are most common around peak height velocity (i.e. the fastest period of somatic growth), the incidence/burden of lower-limb soft-tissue injury increases with advancing maturity status, i.e. post-PHV > circa-PHV > pre-PHV.
Fast growth (an increase in height of more than 7.2 cm per year) appears to increase the risk of injury in male academy football players.
All studies reviewed were conducted in *male* academy football players and, given the sex differences in biological maturation and injury risk, future research should investigate the association of growth and maturation with injury in *female* academy players.

## Introduction

Football (soccer) is the most popular sport worldwide and is played by men, women, boys and girls at a range of competitive standards [1]. Players who reach the professional and elite levels of the sport have often participated from a young age and may have undergone specific training within youth academies of professional football clubs. Even though just ~ 13% of talented youngsters eventually play professional football, these youth football academies follow strategies tasked with finding and developing promising young players through specialised programmes to perfect their playing ability [2].

All adult players undergo biological maturation in their teenage years, where considerable somatic changes occur in preparation for adulthood. The point of fastest somatic growth during puberty is termed ‘peak height velocity’ (PHV), and although girls and boys tend to undergo PHV around 11.5 and 13.5 years, respectively [3], the chronological age at which PHV occurs varies considerably [4]. During the developmental years spent by young players in football academies, categorisation is predominantly according to their chronological age. Consequently, boys and girls within single-year age groups who are advanced in maturity are, on average, taller and heavier than those who are yet to mature physically [4, 5]. Among several proposed consequences of these somatic differences is the suggestion that some academy players may be at an increased risk of injury because of rapid growth [6], or the potential size mismatch that can exist between chronologically age-matched players [7].

Injury is the primary factor influencing player availability in professional football [8] and affects the development of youth players [9]. It is possible that the anthropometric differences due to variation in biological maturation might impact academy players’ tolerance to training loads and their risk of injury, as might the growth process itself. This theory has given rise to an exponential increase in the number of studies in the last 5–10 years, investigating an association between biological maturity and injury risk in academy football players. However, these studies report equivocal conclusions, potentially influenced by the fact that not all studies adopt the same definitions, measures and/or methodologies with respect to both biological maturity and injury reporting. For example, the most reported measures of maturity in the context of injury are maturity *timing* and *status*. Despite being independent variables, the terms are often used interchangeably. Maturity timing considers the chronological age at which maturity-associated events occur relative to the typical age they are expected to occur, thereby determining whether that player has reached that indicator of maturity early, late or on time. In contrast, maturity status describes an individual’s stage of maturation at the time of measurement. In some instances, where PHV is estimated, maturity status specifies whether an individual is pre-, circa- or post-PHV at a specific point in time. However, alternate methods of assessing the stage of maturation are also available and can include measurement of skeletal age, use of hormone assays and assessing secondary sex characteristics. Another (less researched) approach is to assess the impact of growth rate on injury. This refers to the speed at which a player grows, rather than their maturity status or the age at which they reach PHV.

In recognition of the significant increase in the number of published articles within this field, and the equivocal results reported, we sought to review the literature concerning the association of growth and maturation with injury in youth academy football players. The objectives of this narrative review were (i) to perform a structured and reproducible literature search for studies investigating the link between maturity timing/maturity status/growth rate and injury in youth players; (ii) to objectively evaluate the methodology of eligible studies to highlight best practice and propose areas for improvement in future studies; (iii) to discuss how methodological differences might contribute to discrepant conclusions in a narrative context; and (iv) to propose directions for future research in this field and to provide information for applied practitioners attempting to mitigate injury risk in this population.

## Methods

### Literature Search

**Fig. 1:**
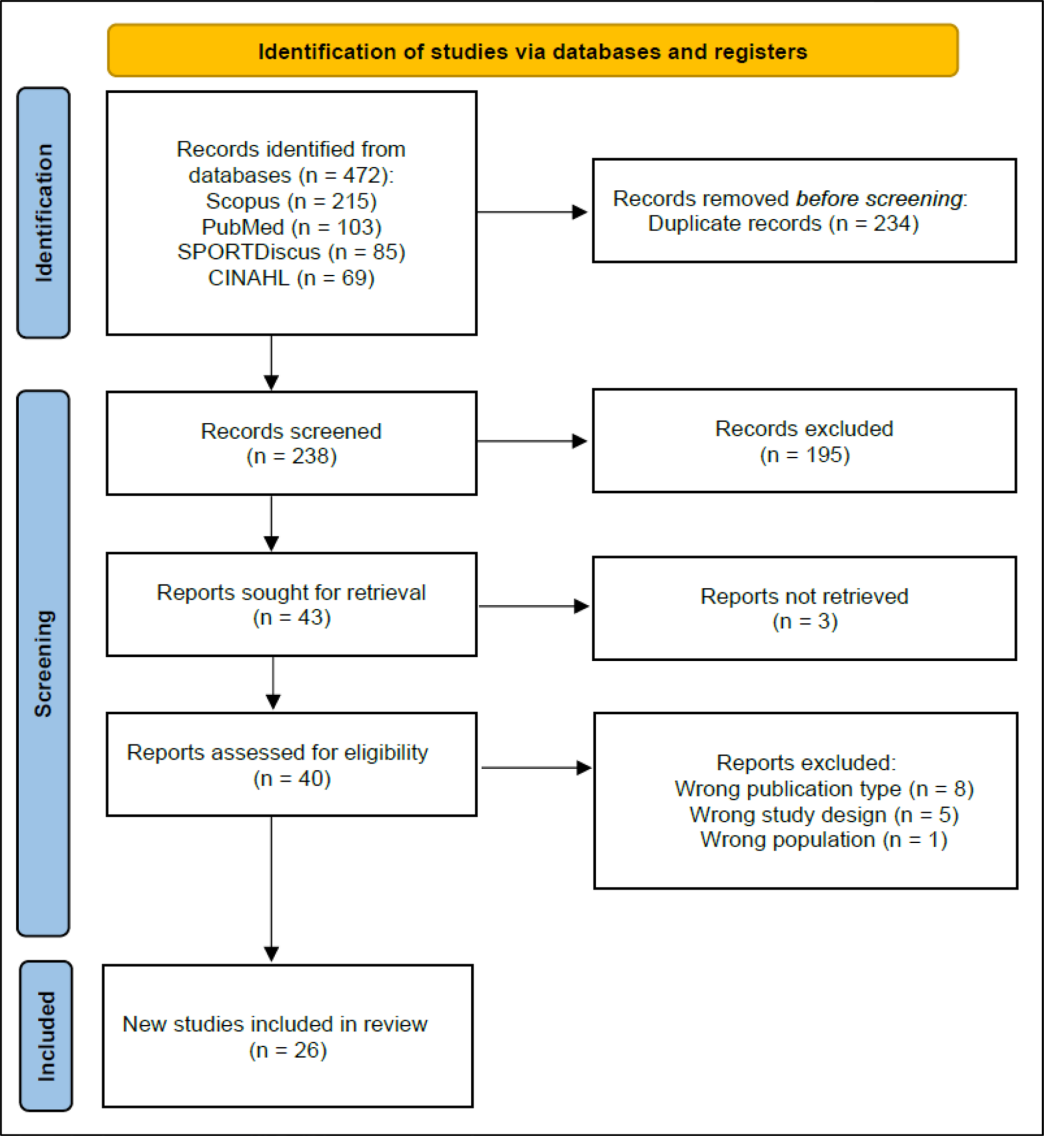
PRISMA flowchart for current literature search

A literature search was conducted for original research studies published up to and including 10 April 2025 using PubMed, Scopus, CINAHL and SPORTDiscus databases. Key terms were searched for within article titles, abstracts, and keywords using conjunctions ‘OR’ and ‘AND’ with truncation ‘*’. Search terms included combinations of the following Boolean phrases: ‘Soccer’, ‘Football’, ‘Maturation’, ‘Maturity’, ‘Status’, ‘Youth’, ‘Adolescent’, ‘Academy’, ‘Biological’, ‘Peak’, ‘Height’, ‘Velocity’, ‘Growth’, “Tempo”, ‘Injury’, ‘Injuries’ and ‘Risk’. Detailed search strings are provided in Electronic Supplementary Material, Appendix 1. Search results were exported in the Research Information Systems (.ris) format and uploaded to Rayyan, a web-based application [10]. Potential duplicate studies were detected by Rayyan and flagged for author screening, with confirmed duplicates subsequently removed. The remaining studies went through three stages of screening. In stage 1, article titles and abstracts were screened to identify and remove inappropriate studies. Such studies were classified as those that did not contain injury data, did not measure or estimate maturity status/maturity timing/growth rate, or those reporting both variables without investigating whether they were related. In stage 2, article full texts were independently reviewed and assessed against predefined inclusion and exclusion criteria (Table 1) by two reviewers (E.H. and R.E.). Studies where reviewers’ opinions on inclusion differed were discussed until arriving at consensus. Studies that did not meet eligibility criteria were excluded. In stage 3, data and key findings from each remaining study were extracted for synthesis and discussion in the current narrative review (Fig. 1).

**Table 1:** Inclusion and exclusion criteria

Inclusion criteria^a^	
Population	Male or female academy soccer players aged U9–U23 years
Intervention/comparator	Invasive/non-invasive methods to predict or estimate maturity status/maturity timing Serial anthropometric measures to calculate growth rate
Outcome variable(s)	Injury (incidence/burden/prevalence/frequency/severity)
Study design	Longitudinal/cross-sectional, either prospective or retrospective
Study settings	Professional soccer club/national team academies worldwide
Exclusion criteria	
Population	Non-academy players Adolescent athletes competing in other sports Adolescents from general population
Age, years	Players aged < 9 and > 23 years Players competing in non-academy, professional soccer
Study characteristics	Studies not written in English Non-peer reviewed literature Descriptive studies Case reports
Outcome variables	Performance characteristics (e.g. soccer-specific skills) Physiological characteristics (e.g. aerobic capacity, high-speed running, muscle strength) Potential injury risk factors (e.g. limb asymmetry, muscle imbalance, landing mechanics)

^a^Inclusion criteria based on Population, Intervention/Comparator, Outcome variables, Study design, Study settings (PICOSS)

### Data Extraction

Studies were grouped for synthesis according to the independent variable of interest, i.e. the association of maturity timing, maturity status or growth rate with injury. Studies quantifying the impact of more than one of these variables on injury were allocated to multiple groups with data extracted independently for each variable. Article screening and data extraction was performed independently by two researchers. Article information (authors, title, journal and main outcome) for all articles sought for retrieval after title and abstract screening were compiled using Microsoft Excel. Next, data were extracted using a custom template [Electronic Supplementary Material, Appendix 2] by each reviewer independently with any differences discussed and resolved by consensus before quality assessment was conducted. The following data were extracted:

- Publication data: authors, year, title of study, journal
- Study duration and design: length of study (months, years or seasons)
- Countries: name(s) of country/countries players were recruited from
- Number of academies: number of participating academies
- Sample size: number of individual players
- Age range: range of chronological age groups studied
- Maturity-related variable(s): whether the study investigated maturity timing/maturity status/growth rate (or multiple)
- Assessment method(s): method(s) used to assess maturity timing/maturity status/ growth rate (or multiple)
- Grouping variable: how players were categorised according to maturity timing/maturity status/growth rate (or multiple)
- Injury quantification: method(s) used to quantify injury according maturity timing/maturity status/growth rate, and how each was calculated
- Injury classification: details of injury type and/or location analysed according to maturity timing/maturity status/growth rate

### Quality Assessment

The Observational Study Quality Evaluation (OSQE) [11] tool was used to objectively assess study quality. The shortened ‘cross-sectional’ version [Electronic Supplementary Material, Appendix 3] was selected owing to its applicability and assessment of components such as representativeness of the study population, validity of independent and dependent variables, and statistical methods. Whilst quantitative assessment of articles using the OSQE is discouraged [11], the sum of positive scores (stars) can be provided as an outcome measure when evaluating study quality and, consequently, risk of bias. A higher sum of stars indicates better study quality and reduced risk of bias, with a maximum of 9 stars available. Studies assessing combinations of maturity variables were scored independently for each variable. For example, studies assessing both maturity timing and maturity status were scored using the OSQE for maturity timing and separately for maturity status, and were therefore awarded two independent scores.

## Results

### Literature Search

A total of 472 articles were returned, with 234 duplicates removed and 195 articles deemed irrelevant on the basis of title and abstract (Fig. 1). A total of 14 articles were excluded during full text screening (Fig. 1) owing to being the wrong publication type (*n* = 8), wrong study design (*n* = 5) or wrong population (*n* = 1) [Electronic Supplementary Material, Appendix 4]. After full screening, a total of 26 articles met the inclusion criteria and are synthesised in this narrative review. Of these articles, 9 assessed the association between injury and maturity timing (Table 2), 15 assessed the association between injury and maturity status (Table 3) and 9 assessed the association between injury and growth rate (Table 4). Seven articles assessed more than one variable, studying both maturity timing and maturity status (*n* = 2), maturity timing and growth rate (*n* = 1), or maturity status and growth rate (*n* = 4). The eligible studies were published between 2007 and 2025 and described cohorts from academies in England, Spain, Belgium, the Netherlands, Qatar, Italy, France, Brazil, Uruguay and Argentina. Study duration ranged from 9 months to 20 years, and the number of clubs per single study ranged from one to eight, with overall sample sizes ranging from 23 to 501 players. Included studies reported exclusively on male youth players (i.e., no eligible studies recruited female players).

**Table 2:** Studies investigating the association of *maturity timing* with injury in male academy football players

Study	Study duration and design	Country (number of academies studied)	Sample size (age range)	Skeletal age assessment/estimate	Grouping variable	Injury variable(s) assessed	Main findings, strengths and limitations	Quality score (max: 9)
Monasterio et al. (2022) [26]	10 seasons, longitudinal	Basque Country, Spain (1)	*n* = 183 (U14)	Tanner–Whitehouse 2 method [39]	Early (SA > + 0.5 years older than CA) Normal (SA ± 0.5 years of CA) Late (SA > + 0.5 years younger than CA)	Burden (number of days lost per 1000 player training or match hours)	Main findings: Overall burden higher in early versus late Muscle burden higher in early versus on time and late Hamstring burden higher in early versus on time Joint/ligament burden higher in early versus late, on time versus late Ankle burden higher in early versus late Strengths: Measured training and match exposure; radiographic assessment of SA; statistical comparison of specific injury classifications Limitations: Single club/country; single age group only; one season per player	9
Light et al. (2021) [36]	4 seasons, longitudinal	England (1)	*n* = 190 (U13–U16)	Khamis–Roche [17]	*Z*-score based on average maturity timing for U13–U16 players in study Early (*z* > + 1.0) Normal (*z* between − 1.0 and + 1.0) Late (*z* < − 1.0)	Frequency (number of injuries) Incidence (per 1000 h of training and match exposure) Severity (minimal = 1–3 days, mild = 4–7 days, moderate = 1–4 weeks, major = > 4 weeks)	Main findings: No association between maturity timing and injury Strengths: Longitudinal study; individual players measured over consecutive seasons across PHV Limitations: Single club/country; *z*-score derived from study population limits external validity	9
Materne et al. (2021) [31]	4 seasons, longitudinal	Qatar (1)	*n* = 283 (U13–U19)	Fels method [19]	Early (SA > 1 year older than CA) Normal (SA ± 1 year of CA) Late (SA > 1 year younger than CA) Mature (SA = 18 years, fully ossified hand/wrist)	Frequency (number of injuries) Incidence (per 25-player squad season) Burden (days lost per 25-player squad season)	Main findings: Higher hazard ratio for overall injury incidence in early versus normal and mature Lower limb apophyseal injury least common in mature Muscle strain incidence higher in mature versus early and normal Strengths: Longitudinal study; individual players measured across multiple consecutive seasons; radiographic assessment of SA; statistical comparison of specific injury classifications Limitations: Estimated exposure; estimated burden; single club/country	8
Johnson et al. (2009) [30]	6 years, longitudinal	England (1)	*n* = 292 (U9–U16)	Fels method [19]	Early (SA > 1 year older than CA) Normal (SA ± 1 year of CA) Late (SA > 1 year younger than CA)	Incidence (per 1000 h of training and match exposure)	Main findings: Incidence not influenced by maturity timing when adjusted for exposure, height and playing position Maturity timing and exposure (training, match) explained 48% of injury incidence variance Strengths: Longitudinal study; individual players measured across multiple consecutive seasons; measured training and match exposure; radiographic assessment of SA Limitations: Single club/country; statistical comparison of injury types/locations lacking	8
Monasterio et al. (2023) [23]	20 seasons, longitudinal	Basque Country, Spain (1)	*n* = 110 (U11–U19)	Super Imposition by Translation and Rotation (SITAR) model [21]	Age at PHV compared with average of historical Basque population data: Early (< 0.5 years versus average) On time (± 0.5 years versus average) Late (> 0.5 years versus average)	Burden (number of days lost per player season)	Main findings: Overall burden increased with advancing maturity Before PHV, growth and knee joint/ligament less burdensome in late versus on time Late-maturing adult had greater overall and joint/ligament burden than early maturing adult Strengths: Longitudinal study; full academy age range; statistical comparison of specific injury classifications; combines maturity timing and maturity status Limitations: Estimated exposure; single country/club	8
Wik et al. (2024) [32]	3 seasons, longitudinal	Qatar (1)	*n* = 95 (U13–U15)	Fels method [19]	Early (SA > 1 year older than CA) Normal (SA ± 1 year of CA) Late (SA > 1 year younger than CA) Mature (SA = 18 years, fully ossified hand/wrist)	Incidence (per 1000 h training or match exposure) Risk (odds ratio)	Main findings: Older skeletal age associated with risk of sudden onset injury No effects of maturity timing on injury risk Strengths: Longitudinal study; individual players tracked across consecutive seasons around PHV; radiographic assessment of SA Limitations: Single club/country; small sample size; specific injury types/locations not analysed according to maturity timing	8
Le Gall et al. (2007) [25]	10 seasons, longitudinal	France, National Football Institute (1)	*n* = 233 (U14)	Greulich–Pyle [38]	Early (SA > 1 year older than CA) Normal (SA ± 1 year of CA) Late (SA > 1 year younger than CA)	Incidence (based on estimated exposure) Severity (minor = 1–3 days, mild = 4–7 days, moderate = 1–4 weeks, major = > 4 weeks)	Main findings: No significant difference in overall incidence according to maturity timing Higher groin incidence, early and normal versus late Higher tendinopathy incidence, early and normal versus late Higher osteochondral disorder incidence, late and normal versus early Higher incidence of major injuries, late versus early Strengths: Radiographic assessment of SA; statistical comparisons of specific injury classifications Limitations: Estimated exposure; lack of information on injury mechanisms; single club/country	8
Johnson et al. (2020) [34]	2 seasons, longitudinal	England (1)	*n* = 76 (U11–U16)	Khamis–Roche [17]	*Z*-score comparison with Berkeley longitudinal study: Early (*z* > + 0.5) On time/late (*z* < + 0.5)	Incidence (per 1000 h match exposure) Burden (incidence rate × mean days missed per injury)	Main findings: No effect of maturity timing on injury after accounting for maturity status Strengths: Longitudinal study; individual players measured regularly over more than one season; multiple age categories around PHV; combines maturity timing and maturity status Limitations: Non-contact time-loss injuries only; match exposure minutes only; single club/country; small sample size; individual players’ full journey across PHV unlikely to be captured	6
Van der Sluis et al. (2015) [33]	3 years, longitudinal	Netherlands (1)	*n* = 26 (11.9 ± 0.8 years upon entering study)	Maturity offset [16]	Split on the basis of median predicted age at PHV of cohort: Early (age at PHV ≤ 13.91 years) Later (age at PHV ≥ 13.92 years)	Incidence (per 1000 h of training and match exposure) Severity (minimal = 1–3 days, mild = 4–7 days, moderate = 1–4 weeks, severe = > 4 weeks)	Main findings: Higher overuse incidence in later-maturing players before and during period of PHV Strengths: Longitudinal study; individual players tracked across PHV; measured training and match exposure Limitations: Small sample size; single club/country; traumatic versus overuse injuries only; groups players as early versus late maturity timing only (none considered “on time”); narrow age range	6

Quality score derived from evaluating each study against nine specific quality criteria outlined in Electronic Supplementary Material, Appendix 3 (maximum score = 9)

SA, skeletal age; CA, chronological age; PHV, peak height velocity; SITAR, Super Imposition by Translation and Rotation

**Table 3:** Studies investigating the association of *maturity status* with injury in male academy football players

Study	Study duration and design	Country (number of academies studied)	Sample size (age range)	Skeletal age assessment/estimate	Grouping variable	Injury variable(s) assessed	Main findings, strengths and limitations	Quality score (max: 9)
Hall et al. (2022) [29]	1 season, cross-sectional	England (5) Spain (1) Uruguay (1) Brazil (1)	*n* = 501 (U9–U23)	Maturity offset [16]	Pre-PHV (MO ≤ − 1.0); Circa-PHV (MO between − 0.5 and + 0.5); Post-PHV (MO + 1.0 to + 3.5); Adult (MO ≥ + 4.0)^a^	Prevalence (percentage of players injured once or more per injury category) Days missed (return date minus injury date) Incidence (per 1000 h training and match exposure) calculated in a sub-sample (*n* = 166)	Main findings: Higher prevalence circa-, post-PHV and adult versus pre-PHV Higher prevalence post-PHV and adults versus pre for non-contact, soft-tissue, non-contact soft-tissue, muscle, thigh, ankle, hamstring Higher prevalence adult versus pre-PHV for ligament/tendon Higher prevalence circa versus post-PHV for growth-related Incidence patterns similar in sub-sample (*n* = 166) Cumulative days missed to injury higher in post- and adult versus pre-PHV Strengths: Sample size; full academy age range; multiple clubs/countries; statistical comparison of specific injury classifications; correction for error in maturity offset method; distinction between post-PHV and adult Limitations: Single season; single measurement of PHV status	8
Monasterio et al. (2023) [23]	20 years, longitudinal	Basque Country, Spain (1)	*n* = 110 (U11–U19)	SITAR model [21]	Pre-PHV (> 6 months before PHV) Circa-PHV (± 6 months from PHV) Post-PHV (> 6 months after PHV until < 1 cm/year growth velocity) Adult (until leaving academy)	Burden (number of days lost per player season)	Main findings: Overall burden increased with advancing maturity status Overall burden lower in pre- versus circa-, post-PHV and adult Growth-related burden highest in circa-PHV Muscle burden higher for post- and adult versus circa- and pre-PHV Hamstring burden higher in circa-, post- and adult versus pre-PHV Quadriceps burden higher in post- and adult versus pre-PHV Joint/ligament, knee and ankle burden higher in post- and adult versus pre- and circa-PHV Strengths: Longitudinal study; full academy age range; statistical comparison of specific injury classifications; distinction between post-PHV and adult; combines maturity status and maturity timing Limitations: Estimated exposure; comparison with unique population (Basque); single country/club	8
Monasterio et al. (2023) [22]	20 years, longitudinal	Basque Country, Spain (1)	*n* = 124 (U11–U19)	SITAR model [21]	Circa-PHV (± 6 months from PHV) Post-PHV (> 6 months after PHV until < 1 cm/year growth velocity)	Burden (number of days lost per player season)	Main findings: Growth-related burden higher in fast-growing circa-PHV players Strengths: Longitudinal study; individual players measured serially for several seasons; specific injury classifications Limitations: No pre-PHV players reported; estimated exposure; comparison with unique population (Basque); single country/club	8
Monasterio et al. (2024) [24]	20 years, longitudinal	Basque Country, Spain (1)	*n* = 84	Used % of observed adult height	Pre-PHV (< 88%) Circa-PHV (88–95%) Post-PHV (> 95%)	Incidence (per 1000 h training and match exposure) Burden (days lost per 1000 h training and match exposure)	Main findings: Direct comparisons of pre- versus circa- versus post-PHV not presented In pre-PHV, higher incidence and burden in faster growing players versus moderate or slow growth In circa-PHV, higher incidence and burden in faster growing players versus moderate growth In post-PHV, growth-related incidence higher in faster growing players versus slow growth Strengths: Longitudinal study tracking players across development; regular growth measures; data based on final adult height; describes interaction of growth rate and maturity status; statistical comparison of specific injury types/locations Limitations: Single club/country; estimated training and match exposure	8
Bult et al. (2018) [41]	3 seasons, longitudinal	Netherlands (1)	*n* = 170 (U12–U19)	Maturity offset [16]	Pre-PHV groupings: PHV 1 (> 12 months to PHV); PHV 2 (6–12 months to PHV); PHV 3 (< 6 months to PHV) Post-PHV groupings: PHV 4 + 5 (< 3 months to 6 months after PHV); PHV 6 (> 6 months after PHV)	Incidence (per 1000 h training and match exposure) Burden (number of injuries × median days lost per injury, per 1000 h exposure)	Main findings: Higher incidence for PHV 4 + 5 versus overall mean Higher burden for PHV 4 + 5 versus overall mean Strengths: Longitudinal study; serial maturity estimates; measured individual exposure minutes Limitations: Single country/club; not all players contributed multiple seasons; specific injuries not analysed statistically	7
Monasterio et al. (2021) [42]	21 years, longitudinal	Basque Country, Spain (1)	*n* = 63 (U11–U19)	Used % of observed adult height	Pre-PHV (< 88%) Circa-PHV (88–96%) Post-PHV (> 96%)	Frequency (number of injuries)	Main findings: Growth-related most common pre- and circa-PHV^C^ Muscle, knee joint/ligament and ankle joint/ligament most common post-PHV^C^ Strengths: Longitudinal study; individual players measured serially from ≤ U12 to adulthood; specific injury classifications (descriptive) Limitations: Single country/club; small sample size; descriptive injury analysis only	7
Toselli et al. (2021) [49]	1 season, cross-sectional	Italy (1)	*n* = 141	Maturity offset [16]	Pre-PHV (≥ 6 months to ≤ 18 months before PHV) PHV (± 6 months from PHV) Post-PHV (≥ 6 months to ≤ 18 months after PHV)	Incidence (per 1000 h of training and match exposure) Severity (slight = no absence, minimal = 1–3 days, mild = 4–7 days, moderate = 1–4 weeks, severe = > 4 weeks)	Main findings: No significant difference in injury incidence according to PHV classification Days missed higher in PHV versus post-PHV Strengths: Measured training and match exposure Limitations: One season only; single club/country; specific injury types not compared statistically	7
Van der Sluis et al. (2014) [40]	3 years, longitudinal	Netherlands (1)	*n* = 26 (11.9 ± 0.8 years upon entering study)	Maturity offset [16]	Pre-PHV (≥ 6 months to ≤ 18 months before PHV) PHV (± 6 months from PHV) Post-PHV (≥ 6 months to ≤ 18 months after PHV)	Incidence (per 1000 h of training and match exposure) Severity (slight = no absence, minimal = 1–3 days, mild = 4–7 days, moderate = 1–4 weeks, severe = > 4 weeks)	Main findings: Higher number of traumatic injuries in PHV versus pre-PHV Strengths: Longitudinal study; individual players tracker across maturation; measured training and match exposure Limitations: Small sample; single club/country; traumatic versus overuse injuries only; narrow age range	7
Dominguez et al. (2025) [44]	1 season, cross-sectional	Argentina (1)	*n* = 212 (U14 to reserve)	Maturity offset [16]	Pre-PHV (MO ≤ − 1.0) Circa-PHV (MO between − 0.5 and + 0.5) Post-PHV (MO ≥ + 1.0)^*b*^	Incidence (per 1000 h of training and match exposure) Burden (number of days of time loss/1000 h exposure) Severity (0 days; mild = 1–7 days, moderate = 8–28 days, severe = > 28 days)	Main findings: Functional disorder of rectus femoris burden higher for circa-PHV versus post-PHV Post-PHV higher overall incidence, burden, severity versus circa-PHV^*c*^ Strengths: Injuries detailed according to type/location Limitations: Most quantitative data are descriptive; pre-PHV excluded from analysis owing to low numbers (*n* = 3); exposure estimated on squad basis	6
Dut et al. (2023) [50]	18 months, cross-sectional	Turkey (2)	*n* = 206 (13.4 ± 1.5 years upon entering study)	Tanner pubertal stages form (self-report against)	Tanner stages 1–5	Incidence (per 1000 h of training and match exposure)	Main findings: Highest proportion of injuries (overall) in Tanner stage 4 (i.e. around PHV) More players with 2 + injuries (overall) in Tanner stage 4 Strengths: Also investigated influence of growth-rate on injury Limitations: Unclear whether incidence estimated or measured individually; injury details not always recorded by physicians (e.g. reported by players, parents); interaction of Tanner stages with growth rate not analysed; limited data on injury type/location	6
Johnson et al. (2020) [34]	2 seasons, longitudinal	England (1)	*n* = 76 (U11–U16)	Khamis–Roche [17]	Using % of predicted adult height: Pre-PHV (< 88%) Circa-PHV (88–95%) Post-PHV (> 95%)	Incidence (per 1000 h match exposure) Burden (incidence rate × mean days missed per injury)	Main findings: Higher incidence circa- versus pre-PHV Higher burden circa- versus pre-PHV Higher burden post- versus pre-PHV Strengths: Longitudinal study; individual players measured serially across more than one season Limitations: Non-contact time-loss injuries only; match exposure minutes only; single club/country; small sample size	6
Mandorino et al. (2022) [46]	1 season, cross-sectional	Italy (1)	*n* = 23 (U14)	Maturity offset [16]	Pre-PHV (MO ≤ − 0.3) Circa-PHV (MO between − 0.3 and + 0.3) Post-PHV (MO ≥ + 0.3)	Incidence (per 1000 h of training and match exposure) Burden (number of injuries × mean days missed per injury per 1000 h exposure)	Main findings: Higher incidence circa- versus pre-PHV Higher burden circa- versus pre-PHV Higher burden post- versus pre-PHV Strengths: Measured training and match exposure Limitations: Small sample size; single age group; statistical comparison only available for overall injury incidence/burden	6
Robles-Palazon et al. (2022) [43]	9-month season, cross-sectional	Spain (5)	*n* = 314 (U11–U19)	Maturity offset [16]	Pre-PHV (MO ≤ − 1.0) Circa-PHV (MO between − 0.5 and + 0.5) Post-PHV (MO ≥ + 1.0)^b^	Prevalence (percentage of players injured once or more per category) Incidence (per 1000 h of training and match exposure) Burden (days lost per 1000 h of training or match exposure) Severity (slight = no absence, minimal = 1–3 days, mild = 4–7 days, moderate = 1–4 weeks, severe = > 4 weeks)	Main findings: Overall incidence higher for circa- versus pre-PHV Overall incidence higher for post- versus pre-PHV Overall burden highest for circa- versus pre- and post-PHV Overall burden higher for post- versus pre-PHV Traumatic burden higher for post- versus pre-PHV Overuse risk higher for circa- versus pre- and post-PHV Strengths: Sample size; multiple clubs; correction for error in maturity offset method Limitations: Short study duration; estimated training exposure; single country; only traumatic versus overuse injuries analysed according to maturity status	6
Read et al. (2018) [48]	1 season, cross-sectional	England (6)	*n* = 356 (U11–U18)	Maturity offset [16]	Maturity offset (continuous variable) used in regression model	Frequency Severity (slight = 2–3 days, minor = 4–7 days, moderate = 1–4 weeks, severe = > 4 weeks)	Main findings: In U13–U14 only, greater maturity offset associated with increased injury risk Strengths: Sample size; multiple clubs Limitations: Single season; single measurement; injuries not expressed relative to exposure; lack of specific injury categorisation	5
Rinaldo et al. (2021) [47]	1 season, cross-sectional	Italy (1)	*n* = 88 (U9–U13)	Maturity offset [16]	YPHV-0 (MO > − 1.0) YPHV-1 (MO between − 1.0 and − 2.0) YPHV-2 (MO between − 2.0 and − 3.0) YPHV-3 (MO ≤ − 3.0)	Incidence (per 1000 h of training and match exposure)	Main findings: Injured players closer to PHV than non-injured players Limitations: Small sample size; single club; sample predominantly pre-PHV with no post-PHV players; no statistical data on specific injury types/locations	4

Quality score derived from evaluating each study against nine specific quality criteria outlined in Electronic Supplementary Material, Appendix 3 (maximum score = 9)

MO, maturity offset; PHV, peak height velocity; SITAR, Super Imposition by Translation and Rotation

^a^Players recording maturity offset from − 1.0 to − 0.5, 0.5 to 1.0, and 3.5 to 4.0 removed to account for error in the maturity offset method (∼0.5 years)

^b^Players recording maturity offset from − 1.0 to − 0.5 and 0.5 to 1.0 removed to account for error in the maturity offset method (∼0.5 years)

^c^Descriptive (non-statistical) analysis presented

**Table 4:** Studies investigating the association of *growth rate* with injury in male academy football players

Study	Study duration and design	Country (number of academies studied)	Sample size (age range)	Growth rate measurement	Injury variable(s) assessed	Main findings, strengths and limitations	Quality score (max: 9)
Monasterio et al. (2023) [22]	20 years, longitudinal	Basque Country, Spain (1)	*n* = 124 (U11–U19)	SITAR model [21] to estimate growth-rate percentiles in centimetres per year (fast = ≥ 75th: ≥ 10.84 cm/year; average = 25–75th: 8.98–10.84 cm/year; slow = ≤ 25th: ≤ 8.98 cm/year)	Burden (number of days lost per player season)	Main findings: Growth-related burden higher in fast-growing circa-PHV players No association of maturity tempo with injury in post-PHV players Strengths: Longitudinal study tracking players across development; categorised players as circa- and post-PHV to remove confounding; categorised rate of growth; statistical comparison of specific injury classifications Limitations: Estimated exposure; single club/country	8
Monasterio et al. (2024) [24]	20 years, longitudinal	Basque Country, Spain (1)	*n* = 84	Growth velocity (height) in centimetres per year (‘high’ rate = > 7.2 cm/year, ‘moderate’ rate = 7.2–3.5 cm/year; ‘low’ rate = < 3.5 cm/year	Incidence (per 1000 h training and match exposure) Burden (days lost per 1000 h training and match exposure)	Main findings: In pre-PHV, higher incidence and burden in faster growing players versus moderate or slow growth Circa-PHV, higher incidence and burden in faster growing players versus moderate growth In post-PHV, growth-related incidence higher in faster growing players versus slow growth Strengths: Longitudinal study tracking players across development; regular growth measures; data based on final adult height; describes interaction of growth rate and maturity status; statistical comparison of specific injury types/locations Limitations: Single club/country; estimated training and match exposure	8
Wik et al. (2024) [32]	3 seasons, longitudinal	Qatar (1)	*n* = 95 (U13–U15)	Growth velocities (height, weight) in centimetres and kilograms per year, respectively	Incidence (per 1000 h training and match exposure) Risk (odds ratio)	Main findings: Older skeletal age associated with risk of sudden onset injury No effects of maturity tempo on injury risk Strengths: Longitudinal study; individual players tracked across consecutive seasons around PHV; measured training and match exposure Limitations: Sample size; specific injury types/locations not analysed according to growth rate	8
Johnson et al. (2022) [37]	1 season, cross-sectional	England (1)	*n* = 49 (U13–U16)	Growth velocity (height) in centimetres per year (‘high’ rate = > 7.2 cm/year, ‘low’ rate = < 7.2 cm/year based on Kemper et al. [6])	Frequency (number of injuries) Incidence (per 1000 h training and match exposure) Days missed (return date minus injury date) Burden (incidence rate^*^ mean days missed per injury)	Main findings: Linear relationship between growth velocity and injury 1.73 times injury risk per 2 standard deviation increase in growth velocity Players growing > 7.2 cm/year 74% more likely to be injured versus those growing < 7.2 cm/year Peak injury burden observed at growth velocity of 4.17 cm/year Strengths: Measured training and match exposure; serial height measurements across consecutive seasons; combines growth rate with maturity status Limitations: Small sample size; single club/country; non-contact time loss injuries only; no detail on specific injury types/locations	7
Kemper et al. (2015) [6]	1 season, cross-sectional	Netherlands (1)	*n* = 101 (U12–U19)	Growth velocity (height) in centimetres per month BMI change in kg/m^2^ per month	Incidence (per 1000 h training and match exposure) Severity (slight = no absence, minimal = 1–3 days, mild = 4–7 days, moderate = 1–4 weeks, severe = > 4 weeks)	Main findings: 1.73 higher odds of injury if growth ≥ 0.6 cm/month 1.61 higher odds of injury if BMI increase ≥ 0.3 kg/m^2^ Strengths: Several age groups; measured training and match exposure Limitations: Estimated exposure; single club; no statistical comparison of specific injury types/locations according to growth rate	7
Rommers et al. (2020) [52]	1 season, cross-sectional	Belgium (4)	*n* = 314 (U10–U15)	Growth velocities (height, leg length) in centimetres per year	Incidence (number of injuries per player season) Burden (days lost per player season)	Main findings: 62% higher injury risk per 1 cm leg growth (U10–U12 only) 10% lower injury risk per 1 cm increase in height (U13–U15 only) Strengths: Sample size; multiple clubs Limitations: Estimated exposure; statistical comparison of acute versus overuse injuries only; single country	7
Rommers et al. (2021) [53]	6 months, cross-sectional	Belgium (8)	*n* = 378 (U13–U15)	Growth velocity (height) in centimetres per year	Incidence (per 1000 h training and match exposure)	Main findings: 15% higher odds of injury per 1 cm/year increase in height Strengths: Sample size; multiple clubs; measured training and match exposure Limitations: Short study duration; narrow age range; only two stature measurements; statistical comparison of overuse versus traumatic injuries only	7
Dut et al. (2023) [50]	18 months, cross-sectional	Turkey (2)	*n* = 206 (13.4 ± 1.5 years upon entering study)	Growth velocity (height) in centimetres per year Single measure compared with previous height measure (reported by player/parent)	Incidence (per 1000 h of training and match exposure)	Main findings: Higher growth rate standard deviation score (*z*-score) associated with injury Youth players have higher growth rates than age-matched adolescents Strengths: Also investigated influence of Tanner stage (maturity status) on injury Limitations: Both growth measures not taken by study researchers; unclear whether incidence estimated or measured individually; injury details not always recorded by physicians (e.g. reported by players, parents); interaction of Tanner stages with growth rate not analysed; limited data on injury type/location	6
Rinaldo et al. (2021) [47]	1 season, cross-sectional	Italy (1)	*n* = 88 (U9–U13)	Growth velocities (height, leg length) in centimetres per year	Player-reported using questionnaire Incidence (per 1000 h training and match exposure)	Main findings: Higher growth rates for injured versus non-injured players (6.8 versus 5.2 cm per year, respectively) Limitations: Estimated exposure; injury recording based on player recall; age range unlikely to capture peak pubertal growth (i.e. PHV); no statistical data on specific injury types/locations	6

Quality score derived from evaluating each study against nine specific quality criteria outlined in Electronic Supplementary Material, Appendix 3 (maximum score = 9)

BMI, body mass index; MO, maturity offset; PHV, peak height velocity; SITAR, Super Imposition by Translation and Rotation

### Methods of Assessing or Estimating Skeletal Age

Skeletal age can be determined using radiographic measurement, as performed in the Greulich–Pyle, Tanner–Whitehouse and Fels methods [12, 13]. Each involves analysing X-ray images of the left hand and wrist to determine skeletal age by comparing bone development against reference images, with some methods also incorporating the measurement of bone widths or the assessment of sexual development on the basis of secondary sex characteristics. To assess maturity timing, chronological age is compared with the individual’s skeletal age as determined via radiographic methods and/or secondary sex characteristics, establishing whether an adolescent has advanced in maturity at a chronological age considered to be early, on time or late. Several methods are available for assessing skeletal age for the purposes of estimating timing and maturity status, each with limitations. A recent systematic review highlighted poor concordance between invasive and non-invasive methods used for these purposes in academy football players [14], whilst a study comparing methods used to estimate maturity status in similar populations suggested that equations predicting maturity offset will misclassify 30–50% players [15].

Nevertheless, owing to the intrusive nature of assessing secondary sex characteristics in non-clinical situations, and the practical challenges of radiography in academy football environments [16], predictive equations have been frequently used for estimating maturity status. The two most common methods used in studies of youth football players are the ‘maturity offset’ (Mirwald) [16] and ‘percentage of predicted adult height’ (PPAH, or Khamis–Roche) [17] methods, though others exist such as the Moore-1, Moore-2 and Fransen equations [14, 15]. The Mirwald method uses sex-based multiple regression equations incorporating chronological age and anthropometry to calculate ‘maturity offset’, estimating age in years from the estimated age at PHV. The estimate of whether an individual is pre- or post-PHV is based on a negative or positive maturity offset value, respectively. Typically, those aged ± 0.5 years from their estimated age at PHV are considered “circa” PHV, whilst those aged < 0.5 or > 0.5 years are deemed ‘pre’ or ‘post’ PHV, respectively, though various cut-off values are evident within the literature (Table 3). The error in determining maturity offset around PHV is around 0.5 years [16], however this approach performs particularly poorly when predicting age at PHV in early or late-maturing boys and girls compared with those maturing on time, with variability of up to 2 years as children progress further from their age at PHV [18]. The PPAH method [17] was developed using data from the Fels Longitudinal Study [19] and combines chronological age, height and weight with the height of both biological parents to predict the player’s adult height. From this, thresholds according to PPAH are used to determine maturity status. However, a recent analysis concluded that predicted adult height is an inconsistent statistic for determining growth stage [20], and an evident limitation of the PPAH [17] is the need to include both biological parents’ height, which is not always possible. Recently, the Super Imposition by Translation And Rotation (SITAR) model developed by Cole and colleagues [21], has been used to determine PHV in two studies of male academy football players [22, 23]. The SITAR model is a statistical method for assessing and analysing pubertal growth and is designed to capture and explain complex growth patterns. This is achieved through the superimposition of individual growth curves onto a reference curve, accounting for differences in the timing of growth spurts and for variability in the rate of growth relative to the reference curve. Where longitudinal height records are available, the SITAR model appears to better estimate maturity status than the PPAH and Mirwald methods [15]. If available, it is favourable to conduct retrospective analysis on the basis of the percentage of *observed* adult height in players who have achieved full adult stature, as demonstrated in the recent work by Monasterio and colleagues [24], where serial height measurements over several years allowed determination of PHV rather than the use of estimates, which is common among other studies. However, serial measurements that track players across youth through to adulthood are extremely rare and challenging to collect, highlighting the unique insight provided by these studies.

### Association of Maturity Timing with Injury

Studies assessing the potential link between maturity timing and injury in academy football players have utilised a range of methods to determine skeletal age (Table 2). The 2007 study by Le Gall and colleagues investigated 233 French male under-14 (U14) players from the National Football Institute across a 10-year period using the Greulich–Pyle skeletal age determination method [25]. When combining training and match exposure, late-maturing players had a higher incidence of severe injuries (> 4 weeks absence) compared with those maturing early, whereas on-time and late maturing players had a higher incidence of osteochondroses, and early and on-time maturing players had a higher incidence of tendinopathies.

A more recent study adopting the Tanner–Whitehouse 2 skeletal age determination method in 183 male U14 players from the Spanish Basque country found that overall injury burden (i.e. number of injuries per 1000 h × days absent) was higher in early maturing compared with late-maturing players [26]. Similar to the observations of Le Gall et al. [25], the pattern of certain injuries differed between early, on-time and late maturing players. Specifically, muscle injuries were most burdensome in early maturing players, with joint/ligament injuries more burdensome in early and on-time-maturing players [26]. From these studies, it appears that injuries related to joints/ligaments and/or tendons may be more problematic for academy players, who mature early or on time. This could be influenced by early maturing players playing more aggressively, covering greater total distances at high speeds [27], or undertaking leadership roles [28]. However, it is more likely that an interaction between maturity timing and maturity status is involved. Specifically, early maturing players becoming more advanced in maturity will be more likely to suffer injuries that demonstrate a higher incidence with advancing maturity, such as soft-tissue injuries involving skeletal muscle, ligaments and tendons [23, 29]. Interestingly, however, there was no difference in growth injury burden between players maturing early, on time or late in the Basque cohort [26], nor for overall injury incidence in the French cohort [25].

A 6-year study of 9–16-year-old male players from an English academy utilising the Fels method found no difference in injury incidence between early, on-time and late-maturing players [30], though less than half of the cohort remained in the study for more than two seasons. The reasons for the disparities between these three studies [25, 26, 30] may be due to variation in methodologies. For example, three different methods were used to determine skeletal age: (1) Fels [30]; (2) Greulich–Pyle [25]; and (3) Tanner–Whitehouse 2 [26], and three different statistical approaches were used to compare injury incidence/burden between maturity timing groups. Furthermore, Monasterio et al. [26] and Le Gall et al. [25] investigated maturity timing in players solely from the U14 age group (the chronological age at which PHV tends to occur in boys) over 9–10 successive years, whilst Johnson et al. [30] included players of a wider age range (9–16 years), which may have confounded any effect of maturity timing by including players of varying maturity status. A more recent study utilising the Fels method found male Qatari players with a skeletal age > 1 year ahead of their chronological age to be at greater overall risk of injury compared with those whose skeletal age was within 1 year of chronological age, or those with a skeletal age of 18 years [31]. A later study of male Qatari players (also using the Fels method) found that older skeletal age was associated with greater risk of sudden onset injury, but that this was not affected by the timing at which that skeletal age was reached [32]. The discrepancy between the results of these latter two studies could be influenced by differences in sample size (*n* = 95 versus *n* = 283), where a smaller sample is less statistically powered, and may include insufficient numbers of injuries for each maturity timing group. Alternatively, it could be due to a discrepancy between the age range of players recruited within each investigation (12–18 years versus 12–15 years), where a broader age range would potentially include more late-maturing players.

Evidence of a link between maturity timing and injury based on methods to estimate somatic maturity is also inconclusive. Using the Mirwald method [16], a 3-year study from a single football academy in the Netherlands tracked 26 male players and defined maturity timing as ‘early’ or ‘late’, on the basis of whether players matured before or after 13.91 years of age, which was the median age at PHV of the study cohort [33]. Late-maturing players had a higher incidence of overuse injuries, both in the year preceding PHV and the year of PHV itself. However, despite the longitudinal design of this study, it is unlikely that a sample of 26 players is sufficiently powered to detect an association of maturity timing with injury risk. This probably explains why players were categorised as either ‘early’ or ‘late’ maturers, with none considered to be maturing on time, and why statistical comparison was only presented for traumatic versus overuse injuries and not by more precise injury type and/or location. A much larger sample size and study duration would be required to accumulate a sufficient quantity of specific injury classifications to make more meaningful conclusions.

A subsequent two-season study found no association of maturity timing (assessed via the PPAH method [17]) with injury incidence in 76 U11 to U16 male players from a single English academy [34]. Despite the sample size being slightly larger than in the study by Van der Sluis et al. [33], it was still modest and probably limited the statistical power required to investigate the association of maturity timing with different types and locations of injury. This may be reflected by the fact that only non-contact time-loss injuries were investigated in this study. This is a broad category, including a wide variety of injury types/locations that could have been investigated separately in a larger cohort. Furthermore, only match exposure was used in this study, which probably overestimated the overall injury incidence in this relatively small cohort, as injury incidence is generally higher during matches than it is in training [35]. Another study in a different English academy (also using the PPAH method [17]) investigated 190 male players across four seasons and found no association between maturity timing and injury occurrence, type or location [36]. Despite including more players than in the abovementioned studies, this study was constrained by a low number of specific injury types/locations once players were stratified into maturity timing categories. However, in a recent study analysing 20 years of data collected from players in the Spanish Basque Country, 110 male players had serial height measurements taken over the duration of the study and were assessed using the SITAR model [23]. Maturity timing (assessed relative to historical data from the Basque male population) was associated with the burden of certain injury types (Table 2). In the pre-PHV period, late-maturing players experienced a lower burden from overall injuries, growth-related injuries, knee joint/ligament injuries and anterior inferior iliac spine injuries than those maturing on time. Once achieving adult stature, however, injury burden was lowest in those who matured at an early age compared with those who had matured on time or late, and joint/ligament injuries were 4.5-fold more burdensome in adults who had matured late versus those who had matured early. It should be noted, however, that injury burden in this study was defined as the number of days lost per player-season, which is less precise than when burden is calculated from injury incidence on the basis of individualised records of training and match minutes. Nevertheless, this study is one of only two [23, 37] identified by our narrative review as having investigated the interaction between maturity timing and maturity status, which is recommended for future research owing to the potential confounding factor of maturity status when investigating the association of maturity timing with injury risk.

The use of approaches ranging from radiography to estimates of somatic maturity demonstrates the range of methodological differences between studies investigating the association between maturity timing and injury, which might contribute to the equivocal conclusions reported. Variability in sample size between studies may also influence the disparate results, with some studies likely underpowered to detect an association between maturity timing and injury. This problem is compounded by different reference cohorts and/or thresholds being used to determine whether players are early, on time or late maturing. For example, Le Gall et al. [25] used the radiographic Greulich–Pyle [38] method to determine skeletal age and categorised early maturing as those with skeletal age > 1 year older than chronological age, on-time maturing as those with skeletal age within 1 year of chronological age and late maturing as those with skeletal age > 1 year younger than chronological age. A similar approach was adopted in three studies using the Fels method [30–32]. In contrast, a study using radiography and the Tanner–Whitehouse 2 [39] method [26] categorised early maturing as skeletal age older than chronological age by more than 0.5 years, on-time maturing as skeletal age within 0.5 years of chronological age and late maturing as skeletal age younger than chronological age by more than 0.5 years. A similar approach to categorisation of early, on-time- and late-maturing players was also adopted in a later study by Monasterio and colleagues using the non-invasive SITAR model [23]. Whilst the same 0.5-year thresholds were used, comparison was made between age at PHV relative to the average age at PHV in reference data from the Basque population, rather than a comparison of players’ skeletal age with their own chronological age, as calculated elsewhere [25, 26, 30–32]. This approach provides improved accuracy in the comparison against typical age at PHV specifically for the Basque population but limits external validity of the findings for other populations owing to the unique recruitment strategies of this Basque academy.

In studies utilising the PPAH method [17], it is common to define maturity timing groups using *z*-scores. However, different studies calculate player-specific *z*-scores against different reference data, such as the Berkeley Longitudinal Study [34] or the average maturity timing of the U13–U16 players within their own study [36]. Although reference values from longitudinal studies permit comparison against existing data sets often modelled on large populations, they may not be representative of youth football cohorts. Similarly, categorising maturity timing on the basis of the average age at PHV from players within a single study may introduce bias and limit the ability to generalise findings to players in other academies with different player recruitment strategies. Differences in *z*-score thresholds also exist, where Johnson et al. [34] defined the early, on-time and late categories using *z*-scores of >  + 0.5, + 0.5 to − 0.5, and <  − 0.5, respectively. In contrast, Light et al. [36] defined the same timing categories using *z*-scores of >  + 1.0, + 1.0 to − 1.0, and <  − 1.0, respectively. Whilst neither study found any influence of maturity timing on injury, this may reflect issues relating to sample size and subsequent stratification into groups of maturity timing (i.e. early, on time, late). Nonetheless, it is unclear which *z*-score thresholds are most appropriate in this context. If applied to the same sample, the broader *z*-score range for players to be classified as ‘on-time’ in the approach by Light et al. [36] would classify more players in this category relative to ‘early’ and ‘late’, compared with the approach by Johnson et al. [34]. Finally, the study of Van der Sluis and colleagues [33] appears to be the only investigation of maturity timing to employ the Mirwald method [16] and the use of a dichotomous early versus late-maturing split (based on the median predicted age at PHV of players in their study).

Of the available evidence, the approach recently presented by Monasterio and colleagues [23] appears to provide the most robust conclusions regarding maturity timing (Table 2). This study registered data over 20 consecutive seasons and provided data on the burden of specific injuries, with early, on-time- and late-maturing players defined using 0.5-year thresholds. The burden of overall injuries, growth-related injuries, knee joint/ligament injuries and anterior inferior iliac spine injuries was lower during the pre-PHV period for players maturing late than those maturing on time. However, once reaching adult stature, joint/ligament injuries were 4.5-fold more burdensome in players who had matured late versus those who had matured early, and injury burden was lower for those who matured early than those who had matured on time or late. On the basis of this study, pre-PHV players who mature at later chronological ages appear to be at lower risk of injury. Once reaching adult stature, those late-maturing players experience higher burden from joint/ligament injuries, whereas their early maturing peers have the lowest injury burden relative to players maturing on time or late.

**Fig. 2:**
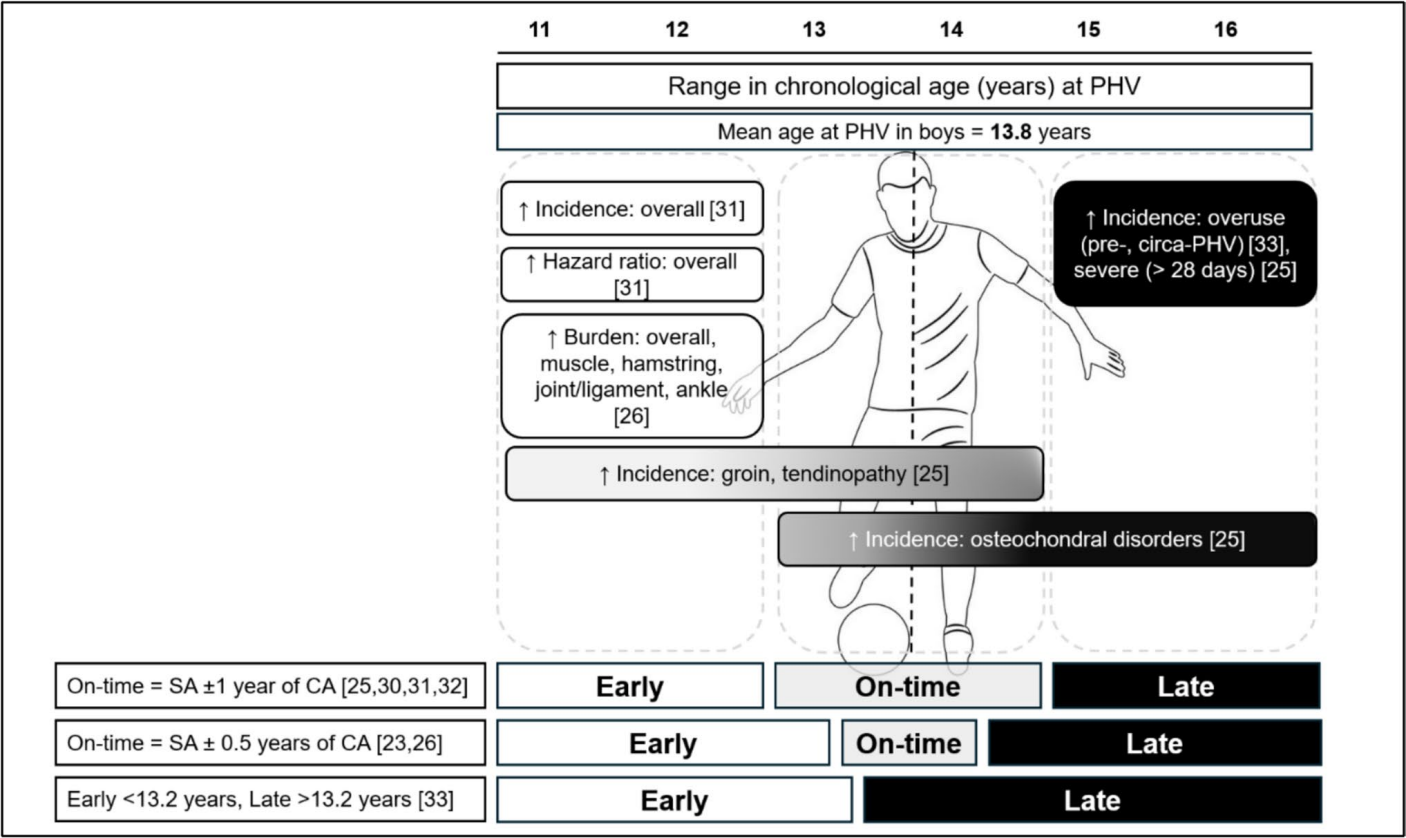
Summary of studies linking injury incidence/burden to maturity timing in male academy footballers. Early (white), on-time (grey) and late (black) boxes represent a variety in thresholds used to define maturity timing on the basis of skeletal age (SA) versus chronological age (CA) difference. Early maturing players were (advanced in maturity) most commonly associated with increased injury incidence/burden. PHV, peak high velocity

As previously outlined, maturity timing is likely to influence the age at which young players become susceptible to injuries associated with advanced maturity, and thus the interaction between maturity timing and maturity status appears to be a key consideration for assessing the impact of maturation on injury in youth football (Fig. 2; Table 2). It is possible that inconsistent conclusions to date are influenced by the fact that most studies have investigated maturity timing or maturity status as single constructs independently, rather than considering both in combination.

### Association of Maturity Status with Injury

A lack of consensus concerning whether maturation influences injury in youth football is also apparent among studies investigating maturity status (Table 3). The previously mentioned study by Van der Sluis et al. [40] estimated PHV using the Mirwald method [16] in 26 male players (aged 11.9 ± 0.84 years) over 3 years. They reported a greater number of traumatic injuries in the year of PHV compared with the preceding year, highlighting growth and maturation as potential injury risk factors. The longitudinal nature of this study is advantageous, yet a small sample from a single academy means statistical comparisons were only possible according to injury severity and whether injuries were traumatic or due to overuse, despite different injury types and locations being recorded. Accordingly, these findings should be interpreted with caution.

A later study of 170 U12–U19-year-old male players from a Dutch academy also used the Mirwald method to investigate injuries across three seasons, though only 58% of players had two or more seasons of injury data available [41]. The researchers adopted a unique approach to PHV classification by establishing six independent PHV periods: three pre-PHV (PHV1 =  > 1 year before PHV; PHV2 = 6–12 months before PHV; PHV3 =  < 6 months before PHV) and three post-PHV (PHV4 =  < 3 months after PHV; PHV5 = 3–6 months after PHV; PHV6 =  > 6 months after PHV). When comparing the *overall* injury incidence in these specific PHV periods against the *overall* injury incidence for all injuries and all players in the study, injury incidence was highest when combining PHV periods 4 and 5 (< 3 months and 3–6 months after PHV, respectively) and lowest when players were > 12 months pre-PHV, with injury burden also highest in the 6 months post-PHV. Crucially, however, and similarly to Van der Sluis et al. [40], the authors did not consider the error in the Mirwald method. Had they done so, their sample size would have been significantly reduced (due to the removal of players in the 1.0–0.5 years pre-PHV and 0.5–1.0 years post-PHV periods) and their conclusions may have been quite different. Furthermore, whilst injury types and locations were reported descriptively, statistical analysis comparing specific injuries between PHV groups was not presented, suggesting that the study lacked statistical power to detect differences between the six PHV classifications. Notably, this study calculated injury burden as the *number of injuries* × *median number of time-loss days per injury per 1000 h of exposure*, whereas others define injury burden as the *number of days lost/1000 h of exposure* [26], the *number of days lost/player-season* [22], or the *injury incidence rate* × *mean days missed per injury* [34, 37]. Inconsistency in the definition and calculation of injury burden limits the ability to make meaningful comparisons between these studies. Where possible, the approach of Bult and colleagues [41] is preferable but relies on accurate exposure data being available for all players from both training and matches. This is not always feasible, particularly in retrospective analyses, and may explain the different approaches evident across studies.

Another cross-sectional study of 76 male players from a single English academy across two seasons determined maturity status using the PPAH method [34]. Injury incidence, risk and burden were all higher in circa-PHV compared with pre-PHV players, though injury burden was greater for post- relative to pre-PHV players. A single, broad category of non-contact time-loss injuries was investigated, rather than specific types or locations of injury, which likely reflects the modest sample size (limiting statistical power), whilst only match exposure minutes contributed to exposure recording, which probably overestimates both injury incidence and burden through the omission of training exposure. A separate *longitudinal* study of 63 male Spanish Basque players found a greater number of injuries in the post-PHV period, though the pattern of distinct injury types differed according to maturity status [42]. Although sample size was once again a limitation, the retrospective nature of this study (using data captured between 1998–2019) enabled the same group of players to be tracked from pre-PHV (U12 age group) to adult stature. Using *measured* (as opposed to estimated) adult height, together with regular stature measurements throughout maturation, maturity status was determined non-invasively with more accuracy than cross-sectional studies that estimate adult height or maturity offset using regression equations. Descriptive analysis revealed that growth-related injuries were most common pre- and circa-PHV, whereas muscle injuries and injuries to the knee and ankle joints/ligaments were more common post-PHV. A more recent study by the same investigators reported the combined association of maturity status and growth rate with injury but did not make direct comparisons between maturity status groups [24]. Nevertheless, this study of 84 male academy players also categorised pre-, circa- and post-PHV players retrospectively on the basis of *measured* adult height, which is a significant strength of both studies [24, 42].

A cross-sectional study including 501 male players from eight football academies in England, Spain, Uruguay and Brazil [29] adopted a similar strategy to Monasterio et al. [42] by including an ‘adult’ group to separate post-PHV academy players who had achieved full adult stature. They reported higher injury prevalence in post-PHV and adult players compared with pre- and circa-PHV players, which was a finding reflected by the injury incidence data in a sub-sample of 166 players with complete exposure data. Similar to the findings of Monasterio et al. [42] relating to injury burden, this study also reported different types of injury being more common at different stages of maturity (Table 3), with a general pattern of greater injury prevalence and incidence with advancing maturity status. This study performed statistical comparisons of specific injury types and locations, offering greater insight into which injuries those working with academy football players might observe as players progress through maturation towards adulthood. This study attempted to minimise any confounding effect of the documented error in the Mirwald equation [16] by removing players with a maturity offset between − 1.0 and − 0.5 or 0.5 and 1.0. Although injury incidence was limited to a sub-sample, *n* = 166 is still larger than most studies investigating the association of maturity status on injury incidence in academy footballers. A subsequent study of 101 players from a Spanish academy across a 9-month season mirrored the approach of Hall et al. [29] in trying to address the error in the Mirwald method and found higher overall injury incidence and burden in circa- compared with pre- and post-PHV, as well as higher incidence and burden in post- than pre-PHV [43]. In this study, traumatic injuries were more common in post- than pre-PHV, with overuse injuries appearing more common circa-PHV than pre- and post-PHV. It is not clear why overall injury incidence and burden were highest in circa-PHV in this study, though the greater number of overuse injuries around circa-PHV probably includes growth-related injuries, because these were not categorised separately as they have been elsewhere [23, 24, 29, 42]. This is a limitation of the study by Robles-Palazon et al. [43], because growth-related injuries are particularly relevant to studies assessing growth and maturation in academy football, with another limitation being the estimation of training exposure rather than actual measured exposure (e.g. using GPS monitoring). Furthermore, a 9-month study from a single academy limits the number of overall injuries recorded, potentially contributing to differences relative to other studies. Most recently, a study of 212 players in Argentina reported rectus femoris muscle injuries as statistically higher in circa- versus post-PHV, using the Mirwald method to determine maturity status [44]. This study also removed players with a maturity offset between − 1.0 and − 0.5, and between 0.5 and 1.0. However, their main conclusions that post-PHV players had higher overall injury incidence, burden and severity were based on descriptive analyses only. Furthermore, only players from the U14 age category and above were recruited, and the small pre-PHV group (*n* = 3) was removed from analysis, limiting the ability to properly investigate the impact of maturity status on injury.

A longitudinal, single academy study spanning two decades tracked 110 male academy players and used the SITAR model to estimate age at PHV [23]. This investigation also found a general pattern of increasing injury with advancing maturity status, with certain injury types more burdensome at specific stages of maturation. Growth-related injuries were more burdensome circa-PHV, whereas injuries to skeletal muscle, joints/ligaments, the hamstrings, the knee and the ankle were each more burdensome in skeletally mature players, mirroring the pattern for injury prevalence and incidence reported by others [29, 43]. A 2022 study from an Italian academy describes overall injury incidence and burden as being higher in circa-PHV players relative to pre-PHV, though this was based on a sample of only 23 players, limiting the ability to provide statistically powered comparisons of different injury types [46]. A further study in an Italian academy investigated players from the U9 to U13 age groups only, reporting that injured players were closer to their estimated age at PHV than non-injured players [47]. However, the lack of older age categories means that the study did not provide a comparison of pre-versus circa-versus post-PHV players, and information on specific injury types was not provided. Moreover, injury reporting was conducted by questionnaires completed by players, which may be less reliable than injuries diagnosed by medical professionals.

It is important to note that not all studies find specific PHV periods to associate with injury. One study reported a relationship of maturity offset with injury that was only evident in players of the U13 and U14 age groups [48], where injured players were further from their estimated age at PHV than non-injured players. It should be noted that this study was an investigation of multiple prospective injury risk factors in academy players using regression analysis, rather than focussing solely on the impact of maturity status. Another study from an Italian academy using the Mirwald method found no impact of PHV group on injury incidence, but observed that circa-PHV players missed more days due to injury than pre-PHV players [49]. Finally, a study of two Turkish academies found that the greatest proportion of injuries were suffered by players in Tanner stage 4, where suffering two or more injuries was also most common [50]. The onset of Tanner stage 4 has been shown to range from 13.4 to 14.4 years in boys [51]. This overlaps the age at which boys typically reach PHV [3, 33], suggesting the players injured most often in that study were likely circa-PHV. Nonetheless, these findings were for injuries overall, and no difference was found between Tanner stages when stratified by injury type. Further investigations are required to determine whether circa-PHV players suffer a greater proportion of injuries, more severe injuries causing longer absences or whether coaches cognisant of the potential effects of PHV on injury deliberately allow additional recovery time for players in age groups where PHV typically occurs.

Despite the Mirwald method [16] being most commonly used to estimate somatic maturation, other methodological discrepancies make between-study comparisons challenging. Furthermore, it is possible that the results from studies using the Mirwald method may have been impacted by the ~ 0.5-year error associated with the Mirwald method [16]. Accordingly, Hall et al. [29], Robles-Palazon et al. [43] and Dominguez et al. [44] all made efforts to account for this error by removing players with maturity offset values in the ranges of − 1.0 to − 0.5 and 0.5 to 1.0, leaving clear distinction between maturity groups. These studies therefore defined pre-PHV as maturity offset less than − 1 year, circa-PHV as maturity offset between − 0.5 and + 0.5, and post-PHV as maturity offset greater than + 1 year. Hall and colleagues also included adult academy players as those with maturity offset greater than + 4.0, after removing those with maturity offset between + 3.5 and + 4.0 to clearly distinguish between ‘post-PHV’ and ‘adult’ players [29]. It should be noted that removing players within these 0.5-year bands is feasible in research studies; however, this approach is impractical in the applied setting, highlighting a limitation of the Mirwald method for practitioners working with youth football players.

The PPAH method and SITAR model have also been used to categorise players as pre-, circa- and post-PHV. Two studies using the PPAH method separated players by maturity status using thresholds according to their PPAH, with minor differences in the percentage values chosen. Johnson et al. [34] defined pre-PHV as < 88%, circa-PHV as between 88 and 95%, and post-PHV as > 95%. This was marginally different from Monasterio et al. [42], who also defined pre-PHV as < 88% but stipulated circa-PHV as 88–96% and post-PHV as > 96%. It is not clear why the boundary between circa- and post-PHV differed in these studies and, whilst it has not been compared statistically, we speculate that this discrepancy would amount to a negligible difference, particularly in studies with limited sample sizes. Two investigations from an academy in the Spanish Basque Country used the SITAR model to address further research questions regarding maturity status using 20 years of longitudinal data. The first categorised players as pre-PHV, circa-PHV, post-PHV or adult to investigate the burden of injury in players who progressed through the academy [23] and found a greater burden of growth-related injuries circa-PHV, yet an increase in overall injury burden was evident as players progressed from pre-PHV through to adults, with this pattern specifically evident for muscle and ligament/joint injuries. The second study focussed on whether injury burden in circa- and post-PHV players was linked to an interaction with growth rate [22] and found that circa-PHV players with fast growth around PHV had the highest overall injury burden and burden from growth-related injuries. Notably, this study investigated the interaction of maturity status and growth rate, categorising players according to their growth rate percentile as fast (≥ 75th percentile: ≥ 10.84 cm/year), average (25–75th percentile: 8.98–10.84 cm/year) or slow (≤ 25th percentile: ≤ 8.98 cm/year), which appears unique to this study.

**Fig. 3:**
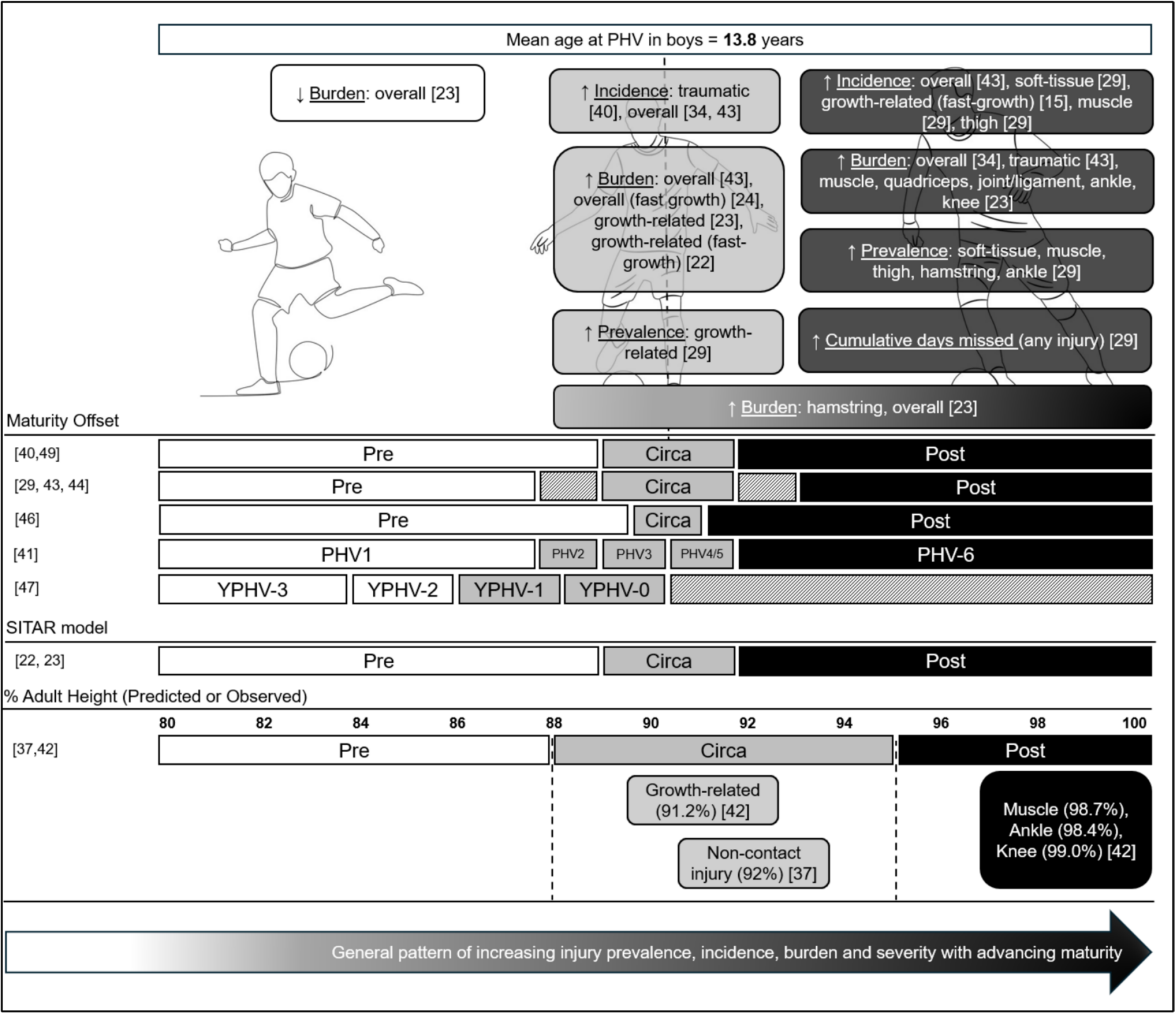
Summary of studies linking injury to maturity status in male academy footballers. Pre (white), circa (grey) and post (black) PHV boxes represent variety in methods and categorisation (maturity offset; % adult height, either predicted or observed; SITAR model) used to estimate maturity status. Post-PHV players were (advanced in maturity) most commonly associated with increased injury incidence/burden. PHV, peak height velocity; YPHV, years from PHV; SITAR, Super Imposition Translation and Rotation

On the basis of the most scientifically robust studies, the strongest evidence suggests that the overall incidence and burden of injury increases with advancing maturity status towards adulthood, with the exception of growth-related injuries appearing most problematic in circa-PHV players undergoing the fastest growth rate [23, 29] (Table 3; Fig. 3). In contrast, the greater incidence and burden of other injuries in physically mature players is likely to reflect the increase in training loads, greater competitive demands observed with advancing age, more aggressive/powerful play linked to an increase in lean mass following puberty [7], as well as the fact that older players are more likely to have suffered previous injury, a known risk factor for injury in football [8]. Whilst two of the most robust studies of maturity status and injury identified in this narrative review differ in duration [23, 29] (Table 3), both provided data on specific injury types and locations, including a separate category for growth-related injuries that is explicitly important for studies in this area. Furthermore, both studies attempted to separate biologically older and younger post-PHV players by including an adult category, allowing for distinction between youth players who had surpassed yet were still close to PHV from those who had reached adult stature but still part of the academies. Future investigations should seek to incorporate these methodological considerations into their study design to provide improved insight into the types of injuries that are most likely for players of different maturity status.

### Association of Growth Rate with Injury

Another approach to investigate the link between the process of biological maturation and injury in football is to measure players’ growth rate, which involves two or more measurements over a fixed period to determine growth velocity. Relative to maturity status, there are fewer studies reporting the impact of growth rate (Table 4). Rommers and colleagues investigated 314 male players (U10–U15) from four Belgian academies and evaluated the impact of growth rate in younger and older players within this cohort [52]. Across one season, U10–U12 players experienced a 62% higher injury risk per 1 cm increase in leg length, whilst injury risk decreased in U13–U15 players with every 1 cm increase in height. However, lack of individual exposure records or information of specific injury types limits the ability to determine whether these injuries were growth related, or the degree to which training load may have influenced players during periods of growth. A second study by the same authors calculated growth rate in 378 male players (U13–U15) from eight Belgian academies using two measurements taken 3–4 months apart during the pre-season, before recording injuries in the first 2 months of the competitive season to investigate whether in-season injuries were associated with growth rates during the preceding pre-season period [53]. They concluded that there was a 15% increase in injury risk for every 1 cm growth per year, which they extrapolated to 2.31 greater odds of injury in a player growing 6 cm per year. This referred to all medical attention injuries (acute and overuse combined) and thus does not imply any specific type or location of injury that may be more likely after periods of accelerated growth. Despite quantifying growth rate, the proportion of pre-, circa- and post-PHV players was not reported. In a U13–U15 cohort, not all players would be circa-PHV, potentially confounding the results, because circa-PHV players would be expected to grow faster than their pre- and post-PHV peers.

Using a different methodology, Johnson and colleagues [37] reported a linear relationship between growth rate and likelihood of non-contact time-loss injuries of unspecified type/location in 49 U13–U16 male players, a cohort that was also likely to have included pre-, circa and post-PHV players. With three to five measurements taken per player per year, those with a high growth rate (> 7.2 cm/year) had greater likelihood of injury than those with a low growth rate (< 7.2 cm/year), whilst there was also a non-linear relationship between growth rate and injury burden. However, this study only considered players to have high or low growth rate, indicating that no players in the study grew at a normal rate. This likely reflects a modest sample size being studied over a single season, which potentially also explains why injuries were not stratified into types and/or locations, and why training and match injuries were not reported separately despite exposure from both settings being recorded. Nevertheless, an earlier study of 101 male players (aged 11–19 years) from a Dutch academy agreed with their findings, concluding that players growing at least 0.6 cm per month were more likely to suffer an injury, whilst a monthly change in body mass index (BMI) (increase > 0.3 kg/m^2^ or decrease > 0.4 kg/m^2^) was also associated with injury occurrence [6]. Whilst not measured, this study probably also included pre-, circa- and post-PHV players, who will exhibit different growth rates as a result. Furthermore, the risk of injury in this study also only referred to overall injury occurrence rather than specific types or locations, which is likely due to small numbers when stratifying injuries into specific categories because of the modest sample size and study duration. Conversely, a recent study by Monasterio and colleagues incorporated longitudinal height records of 124 male players collected across a 20-year period to investigate the combination of maturity status and growth rate during the circa- and post-PHV periods using the SITAR model [22]. Players’ growth per year was categorised as fast (≥ 10.84 cm/year), average (8.98–10.84 cm/year) or slow (≤ 8.98 cm/year) on the basis of percentiles derived from the study cohort, thresholds that are in contrast to those used in the study of Johnson et al. [37], who categorised players as having either a high or low growth rate only. In the circa-PHV period, fast-maturing players had a higher burden from overall and growth-related injuries relative to their average- and slow-growing counterparts. Growth rate had no association with injury burden in post-PHV players. This study did include some injuries according to location (muscle injuries and joint/ligament injuries) and reported no influence of PHV on the burden from those injuries, but the investigation of a single academy is the key limitation of this and most studies in this area.

In a recent study from two Turkish Super League academies, Dut et al. [50] reported that injured players had a higher growth rate *z*-score than non-injured players, and that the same factor was a significant predictor of injury in a logistic regression model. Despite supporting other studies reporting a relationship between growth rate and injury, this was analysed on a binary injured versus non-injured basis, limiting the ability to determine which injury types are most impacted by growth. Growth rate was also assessed by measuring stature and comparing this against player- or parent-reported recall of data from 1 year prior, which may introduce bias/error. Wik and colleagues [32] recently found suggestive evidence that changes in body mass might affect the risk of sudden onset injuries but did not find any effect of increasing height. In 95 male academy players (U13, U14 and U15) from Qatar, the authors investigated somatic growth over intervals on the basis of academy semesters within the football season. Specifically, players were assessed between season start and mid-season and/or from mid-season to the end of the season, leading to 223 growth intervals from the cohort of 95 players, with each interval averaging 113 days in length. Significant associations were found between greater changes in body mass and older skeletal age, and the risk of sudden onset injury, but the authors correctly highlighted the need for larger sample sizes and more frequent anthropometric measurement points to detect growth with greater accuracy. In U9–U13 players from an Italian academy, injured players had higher growth rates than non-injured players across one season (6.8 versus 5.2 cm per year, respectively). However, information was not provided on specific injury types or locations, meaning it is only possible to speculate whether players’ injuries were growth related, and we suggest that not including older age categories means that many players undergoing peak growth may have been missed. Nevertheless, this study adds further weight to the evidence base regarding somatic growth and injury risk in academy footballers.

**Fig. 4:**
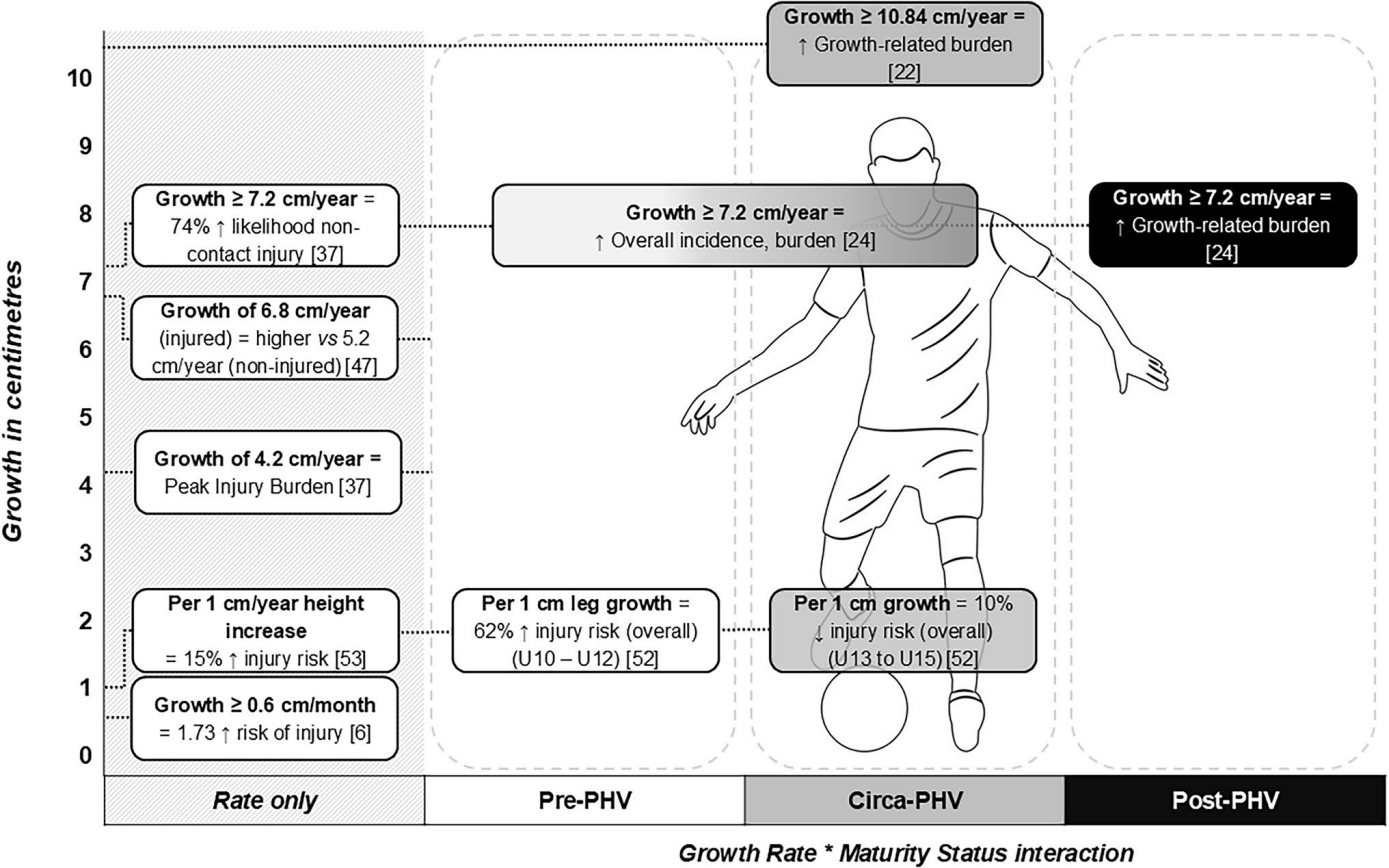
Summary of studies linking injury to growth rate in male academy footballers. *Rate only* details studies assessing growth rate without stratification by maturity status. Pre-PHV (white), circa-PHV (grey) and post-PHV (black) detail studies assessing interaction of growth rate with maturity status. Growth rates ≥ 7.2 cm/year were (i.e. ≥ 0.6 cm/month) most commonly associated with increased injury incidence/burden. PHV, peak height velocity

Whilst there are fewer published studies assessing how growth rate impacts injury in academy football, most available evidence suggests that rapid anthropometric change is associated with an increased risk of injury in this population (Table 4; Fig. 4). This pattern persists despite different methods used to categorise players’ growth rates, variability in sample sizes and considerable discrepancy in the length of time players are monitored, ranging from a few months [53] to decades [22]. For this reason, it may be more appropriate to monitor players’ growth rate rather than estimating their maturity status or percentage of predicted adult stature in studies exploring the link between biological maturation and injury. Indeed, two of the most rigorous studies identified by our quality evaluation [22, 24] not only investigated growth rate but also how different growth rates impact injury in players of different maturity status (Table 4). Furthermore, these studies are among several to categorise growth rates using specific thresholds reflecting growth per year, which practitioners can use to easily identify those who may be at increased risk of injury (Fig. 4).

### Aligning Injury to Percentage of Adult Stature

One retrospective longitudinal study has attempted to discern the precise percentage of adult height where injuries most commonly occur by relating height during childhood to actual *measured* adult height, as opposed to using predicted adult height to create thresholds for maturity status categories. Monasterio et al. [42] reported the median percentage of adult height for growth-related injuries was 91.2%, falling within the circa-PHV grouping in their study. They also reported the median percentages of adult height where muscle injuries, joint/ligament injuries of the ankle and joint/ligament injuries of the knee occurred as 98.7%, 98.4% and 99.0%, respectively, and all of which fell within the post-PHV period, in line with their findings on maturity status (Table 3). Although the data for each player covered the period from U12 (or younger) until adult height, which is a considerable strength relative to other investigations, the sample size of *n* = 63 male players from a single academy remains a limitation.

Using the PPAH method [17], Johnson et al. [37] reported that injury risk increased from 83% PPAH until peaking at 92%, before reducing towards the attainment of adult stature in an English football academy. Similarly, injury burden increased from 83% predicted adult stature before peaking at 95% and then reducing until adult stature was reached. However, this study recruited even fewer male players (*n* = 49) and only followed those players for two seasons. Nonetheless, this evidence indicates that injury may be more problematic in the circa-PHV period at approximately 91–92% of predicted adult height. However, Monasterio et al. [42] reported that this was only the case for growth-related injuries, and that muscle and joint/ligament injuries peaked at more advanced statures (considered post-PHV), mirroring data from studies of maturity status [23, 29] (Table 3). In contrast, Johnson et al. [37] reported data on ‘non-contact time-loss injuries’, and specific injury types and locations were not reported. Considering there are only two published investigations reporting on injuries aligned to the percentage of adult stature, further studies are recommended with larger sample sizes (incorporating multiple academies) and aimed at replicating the longitudinal design adopted by Monasterio and colleagues.

## Discussion

The objectives of this narrative review were (i) to perform a structured and reproducible literature search for studies investigating the association of growth and maturation with injury in academy football players; (ii) to objectively evaluate the methodology of eligible studies to highlight best practice and propose areas for improvement in future studies; (iii) to discuss how methodological differences might contribute to equivocal findings between studies; and (iv) to propose directions for future research and provide information for applied practitioners trying to mitigate injury risk in this population. The first three objectives were achieved by (i) methodically and comprehensively screening the scientific literature investigating the association between maturity status/maturity timing/growth rate and injury in youth football players (Fig. 1); (ii) using an independent quality assessment tool to objectively evaluate the quality of each of the eligible studies (Tables 2, 3, 4); and (iii) discussing the strengths and limitations of individual studies in a narrative context to help clarify the confusion surrounding the association of growth and maturation with injury in this population. The fourth and final objective has been achieved in Sect. 5 of this review, where we suggest areas to be addressed in future research and provide guidance for practitioners.

When investigating the association of maturity *timing*, the most rigorous studies found that lower limb soft-tissue injuries are more problematic to early maturing players [26, 31] (Table 2; Fig. 2). One of these studies suggested that this was specifically the case for muscle, hamstring, joint/ligament and ankle injury burden [26]. A shorter longitudinal study also suggested greater overall injury risk in early maturing players [31], but this is not a universal finding [32]. The longest studies [25, 26] both focus on the U14 age group, which most often contains the greatest proportion of players undergoing PHV (by definition, the fastest growing players). Despite their strengths, these studies are constrained by the investigation of a single club or institution. However, this is universal among studies of maturity timing, warranting larger studies recruiting multiple clubs and/or countries to validate the impact of early maturation on soft-tissue injury across other academies.

The most thorough studies assessing the association of maturity *status* with injury indicate that injuries increase with advancing biological age except growth-related injuries, which are most problematic circa-PHV, and that muscle (e.g. quadriceps, hamstring) and ligament/tendon (e.g. related to the knee, ankle) injuries are most impactful in skeletally mature players [23, 29] (Fig. 3; Table 3). This pattern persists across multiple injury descriptors (prevalence, incidence, burden, days absent) even when different methods are used to estimate maturity status (Fig. 3; Table 3). We suggest that this is likely due to the interaction with maturity timing on maturity status, where different ages at PHV can directly contribute to players of equal chronological age being categorised as pre-, circa- or post-PHV. Early maturing players will become circa- and post-PHV at earlier chronological ages than their peers who mature on time or late, and this is likely to increase their susceptibility to injuries associated with advancing maturity.

Fewer studies have assessed *growth rate* than those investigating maturity status, with scarce data on specific injury types. Fast-growing circa-PHV players appear to have significantly higher injury burden than those growing slower [22], with growth rate unrelated to injury in post-PHV players. Other studies have reached similar conclusions relating to growth rate for non-contact time-loss injuries [37] and overall injuries [6, 50, 53] but are probably underpowered to analyse specific injury types/locations. These studies also did not distinguish between players of differing maturity status, meaning an unknown proportion of players in each study will be circa-PHV with faster growth rates than their pre- and post-PHV peers. Collectively, periods with fast growth rates appear to increase injury susceptibility (Fig. 4; Table 4), with some evidence that this is driven by growth-related injuries. This could be confirmed by more rigorous studies (involving larger sample sizes), investigating the association of maturity timing with injury after initially categorising players’ maturity status and recruiting sufficient players to be able to investigate different injury types and locations.

It is prudent to acknowledge some limitations with this narrative review. Firstly, the review process was conducted by two researchers, which may limit the scope for more diverse perspectives that may aid in reducing any inherent biases held by the authors. However, we reduced the influence of subjective bias by objectively evaluating eligible studies using an established and independent quality assessment tool. Secondly, whilst the focus of this narrative review was on the potential association of maturity with injury in football, we recognise that studies of youth athletes from other sports may contain important data on the broader subject area, and that our exclusion criteria limit the ability to speculate whether observations in other sports may have relevance for practitioners in youth football.

## Directions for Future Research and Practical Applications

As is evident from our objective evaluation process, all eligible studies have limitations that can be addressed in future studies. Those studies that recruited the most players were cross-sectional, single-season investigations [23, 29, 43]. Combining the recruitment of multiple clubs in different countries [29], together with the longitudinal player tracking method over a prolonged period [22, 23], would provide an even more comprehensive description of how maturity status affects injury in academy football players. This not only increases external validity but also the available sample of players and quantity of injuries available, providing the statistical power needed for stratification by specific injury types and locations. Where possible, prospective designs are preferred to ensure accurate exposure minutes can be recorded at the individual player level, which is not always possible in retrospective study designs. Prospective designs may also ensure that regular anthropometric and/or radiographic assessments are scheduled for all players across the study period, reducing the likelihood of excluding players owing to missing data.

Despite numerous studies investigating the association of maturity timing, maturity status and/or growth rate with injury in male academy players, there is a void of evidence in female academy players. Several studies address the topic of injury in female academy players [54–59], but we are unaware of any describing injuries according to maturity status, maturity timing or growth rate. Injury patterns between male and female professional players are not identical, with female players particularly susceptible to severe injuries, including those to the anterior cruciate ligament [60, 61]. This variation in injury risk between males and females and the differences in PHV timing between boys and girls [62] highlight that the findings from studies of male academy players should not simply be applied to female players. Consequently, there is an urgent need for rigorously designed studies, investigating whether injuries in female academy football players are associated with maturity timing, maturity status and/or growth rate.

We also recognise that much of the existing literature lacks guidance for practitioners aiming to reduce growth- and maturation-related injuries. This may be because of discrepant findings among studies, but recent efforts to provide practical solutions on the basis of research data should be acknowledged. Johnson and colleagues [63] demonstrated the success of a tailored injury prevention strategy at reducing growth-related injury incidence and burden in players considered ‘high-risk’ due to the presence of growth-related injury symptomology and/or a combination of fast growth (≥ 7.2 cm/year), fast leg growth (≥ 3.6 cm/year) and being circa-PHV. This study, and the most recent work by Monasterio and colleagues [24] provide heat maps to illustrate where the interaction of growth rate with maturity status is most likely to increase injury incidence and burden in youth players. Whilst each study acknowledges the limitations of their data being from modest sample sizes from individual clubs, both observed similar impacts of growth and maturity status on injury, and the use of heat maps may be particularly attractive to practitioners. Specifically, this approach could be used to monitor squads and individual players in the age groups surrounding the typical age at PHV, with the overarching aim of identifying ‘at-risk’ players who are likely to benefit most from interventions targeted at injury prevention. For example, the study by Johnson et al. [63] adopted an approach combining training load modification, training of football-specific skills, strength training, and drills designed to improve balance, coordination and landing patterns, demonstrating a subsequent reduction in injuries for the players at highest risk. Whilst further studies are needed to confirm the effectiveness of such interventions, this type of approach may have potential for mitigating the increased risk of growth-related injury evident in circa-PHV players [23, 29] and specifically those who experience rapid growth [22].

## Conclusions

Since 2007, there has been an exponential rise in the number of research studies investigating the association between injury and growth and maturation in academy football. However, several methodological differences, limitations and statistically underpowered studies have contributed to a lack of clarity surrounding the association of maturity timing, maturity status and/or growth rate with injury susceptibility in this population. On the basis of the most robust available evidence, we suggest that practitioners working with academy players be cognisant that (i) early maturing circa-PHV players demonstrate a higher risk of soft-tissue (e.g. muscle, ligament, tendon) injuries than their peers; (ii) circa-PHV players with faster growth rates relative to their peers (> 7.2 cm/year) experience greater risk of growth-related injuries than those with slower rates of growth; (iii) growth-related injuries are most problematic in the circa-PHV period than any other maturity status; and (iv) the risk of lower limb soft-tissue injury increases with advancing maturity status, with post-PHV and adult players having the highest risk, not circa-PHV, as might be hypothesised. Moreover, no study has yet investigated the association of maturity timing, maturity status or growth rate with injury in female academy players, and owing to the sex differences in maturity timing, injury risk, physiology, biomechanics and football training and match demands, it may be erroneous to assume that the results from studies in male academy players can be used to inform practice in women’s academy football. Thus, rigorous research study designs (e.g. large-scale, longitudinal studies involving multiple clubs/academies across different countries, and training and match exposure recorded at the individual player level) are recommended for future studies in both male and female players.

## Supplementary Information

Below is the link to the electronic supplementary material.

Supplementary file1 (DOCX 16 KB)

Supplementary file2 (DOCX 17 KB)

Supplementary file3 (DOCX 19 KB)

Supplementary file4 (DOCX 20 KB)

## Declarations

### Data availability statement

Data sharing was not applicable for this manuscript as no datasets were generated or analysed.
