# Supplementary material for: The Association of Growth and Maturation with Injury in Academy Soccer Players: A Narrative Review
Source: Sports Med. 2025 Nov 14;56(1):35–79. doi: 10.1007/s40279-025-02340-0 (PMC12913351; doi:10.1007/s40279-025-02340-0)
Supplement: Supplementary file 1 — Supplementary file1 (DOCX 16 KB) [file 40279_2025_2340_MOESM1_ESM.docx]

**PubMed**

(("Soccer"[Title/Abstract] OR "Football"[Title/Abstract]) AND

("Maturation"[Title/Abstract] OR "Maturity"[Title/Abstract] OR "Status"[Title/Abstract]) AND

("Youth"[Title/Abstract] OR "Adolescent"[Title/Abstract] OR "Academy"[Title/Abstract]) AND

("Biological"[Title/Abstract] OR "Peak"[Title/Abstract] OR "Height"[Title/Abstract] OR "Velocity"[Title/Abstract] OR "Growth"[Title/Abstract] OR "Tempo"[Title/Abstract]) AND

("Injury"[Title/Abstract] OR "Injuries"[Title/Abstract] OR "Risk"[Title/Abstract]))

**Scopus**

(TITLE-ABS-KEY (Soccer OR Football) AND

TITLE-ABS-KEY (Maturation OR Maturity OR Status) AND

TITLE-ABS-KEY (Youth OR Adolescent OR Academy) AND

TITLE-ABS-KEY (Biological OR Peak OR Height OR Velocity OR Growth OR Tempo) AND

TITLE-ABS-KEY (Injury OR Injuries OR Risk))

**CINAHL**

(TI Soccer OR TI Football OR AB Soccer OR AB Football OR MW Soccer OR MW Football) AND

(TI Maturation OR TI Maturity OR TI Status OR AB Maturation OR AB Maturity OR AB Status OR MW Maturation OR MW Maturity OR MW Status) AND

(TI Youth OR TI Adolescent OR TI Academy OR AB Youth OR AB Adolescent OR AB Academy OR MW Youth OR MW Adolescent OR MW Academy) AND

(TI Biological OR TI Peak OR TI Height OR TI Velocity OR TI Growth OR TI Tempo OR

AB Biological OR AB Peak OR AB Height OR AB Velocity OR AB Growth OR AB Tempo OR

MW Biological OR MW Peak OR MW Height OR MW Velocity OR MW Growth OR MW Tempo) AND

(TI Injury OR TI Injuries OR TI Risk OR AB Injury OR AB Injuries OR AB Risk OR MW Injury OR MW Injuries OR MW Risk)

**SPORTDiscus**

(TI Soccer OR TI Football OR AB Soccer OR AB Football OR SU Soccer OR SU Football) AND

(TI Maturation OR TI Maturity OR TI Status OR AB Maturation OR AB Maturity OR AB Status OR SU Maturation OR SU Maturity OR SU Status) AND

(TI Youth OR TI Adolescent OR TI Academy OR AB Youth OR AB Adolescent OR AB Academy OR SU Youth OR SU Adolescent OR SU Academy) AND

(TI Biological OR TI Peak OR TI Height OR TI Velocity OR TI Growth OR TI Tempo OR

AB Biological OR AB Peak OR AB Height OR AB Velocity OR AB Growth OR AB Tempo OR

SU Biological OR SU Peak OR SU Height OR SU Velocity OR SU Growth OR SU Tempo) AND

(TI Injury OR TI Injuries OR TI Risk OR AB Injury OR AB Injuries OR AB Risk OR SU Injury OR SU Injuries OR SU Risk)
