# Supplementary material for: The Association of Growth and Maturation with Injury in Academy Soccer Players: A Narrative Review
Source: Sports Med. 2025 Nov 14;56(1):35–79. doi: 10.1007/s40279-025-02340-0 (PMC12913351; doi:10.1007/s40279-025-02340-0)
Supplement: Supplementary file 2 — Supplementary file2 (DOCX 17 KB) [file 40279_2025_2340_MOESM2_ESM.docx]

| Reviewer ______________ | Date ____/____/________ |
| --- | --- |
| **Study Information** | |
| Author (Year): |  |
| Title of study: |  |
| Journal: |  |
| **Duration/Design, Setting, Sample** | |
| Study duration: |  |
| Study design: |  |
| Country/countries (*n =* ): |  |
| Number of academies (*n =* ): |  |
| Sample size (*n* = ) |  |
| Age range of players: |  |
| **Maturity-specific outcome variable(s)** | |
| Maturity related variable(s) studied (e.g. timing, status, growth rate): |  |
| Assessment method(s) for above variable(s):: |  |
| Grouping of players based on above assessment method: |  |
| **Injury reporting** | |
| Injury quantification methods: |  |
| Injury classification details: |  |
| **Strengths and Limitations** | |
| Main strengths: |  |
| Main limitations: |  |
