# Supplementary material for: The Association of Growth and Maturation with Injury in Academy Soccer Players: A Narrative Review
Source: Sports Med. 2025 Nov 14;56(1):35–79. doi: 10.1007/s40279-025-02340-0 (PMC12913351; doi:10.1007/s40279-025-02340-0)
Supplement: Supplementary file 3 — Supplementary file3 (DOCX 19 KB) [file 40279_2025_2340_MOESM3_ESM.docx]

| **Observation Study Quality Evaluation Criterion** | **Interpretation for Maturity Timing/Maturity Status/Growth Rate** |
| --- | --- |
| **Is the sample optimal for both internal validity and representativeness? Take into account all items (only when a, b, c and d obtain a yes (score 1) item 1 receives a star)** | **As described** |
| a) In- and exclusion criteria --> is the internal validity optimal? |  |
| b) In- and exclusion criteria --> is the external validity optimal? |  |
| c) Is selection proces transparent, leading to representative sample? |  |
| d) Taking into account reasons why participants refuse, do you think data are representative? |  |
| **Is the assessment of the main independent variable valid?** | **"Is the method used to estimate maturity timing/maturity status/growth rate valid?"** |
| Yes (*) |  |
| No |  |
| Don’t know |  |
| **Is the presence of the independent variable optimal? (i.e. severity of exposure, compliance, fidelity to intervention, presence of risk factor, discontinuation of exposure)** | **“Is the independent variable assessed serially (i.e multiple time points) allowing injuries to be correctly aligned to maturation/growth”** |
| Yes (*) |  |
| No |  |
| Unknown |  |
| **Is the assessment of the dependent variable valid?** | **"Is the quantification of injury data valid (i.e following consensus statement or published classification system)?"** |
| Yes (*) |  |
| No |  |
| Unknown |  |
| **Is there a conflict of interest? (e.g. funding or connections with pharmacological industry)** | **As described** |
| No (*) |  |
| Yes |  |
| Unknown |  |
| **Does the statistical analysis control for the relevant confounders? This also includes cointerventions** | **“Does the statistical analysis of injury data control for the influence of exposure on an individual player basis?”** |
| Yes (*) |  |
| No |  |
| Unknown |  |
| **Did the reporting of the results follow a protocol? In other words, were only a priory intended analyses reported? As opposed to cherry picking** | **As described** |
| Yes (*) |  |
| No |  |
| Unknown |  |
| **Systematic reviews and CATs: Is sample size sufficient, looking at calculations/explanation provided by the authors?** | **As described** |
| Yes (*) |  |
| No |  |
| Unknown |  |
| **Reporting (score 1 if yes and if all yes, receives a star)** | **As described** |
| Are the objectives clearly stated? |  |
| Are background characteristic provided? |  |
| Are background characteristic provided stratified by the most relevant exposure or treatment? |  |
| Is statistical analysis described? |  |
