# Supplementary material for: The Association of Growth and Maturation with Injury in Academy Soccer Players: A Narrative Review
Source: Sports Med. 2025 Nov 14;56(1):35–79. doi: 10.1007/s40279-025-02340-0 (PMC12913351; doi:10.1007/s40279-025-02340-0)
Supplement: Supplementary file 4 — Supplementary file4 (DOCX 20 KB) [file 40279_2025_2340_MOESM4_ESM.docx]

| **Author**  **(Year)** | **Title and Journal/Source** | **URL** | **Exclusion Reason** |
| --- | --- | --- | --- |
| Wik, E.H  (2023) | “Injuries in elite male youth football and athletics: growth and maturation as potential risk factors (PhD Academy Award)”  *British Journal of Sports Medicine* | https://pubmed.ncbi.nlm.nih.gov/37652666/ | Wrong publication type |
| Morano, P.J  (2003) | “Injury in youth football: prevalence, incidence, and biological risk factors”  *Michigan State University* | N/A | Wrong publication type |
| Price, R.J et al.  (2001) | “The Football Association medical research programme: an audit of injuries in academy youth football”  *British Journal of Sports Medicine* | https://pubmed.ncbi.nlm.nih.gov/15273188/ | Wrong study design |
| Palma, A.  (2009) | Elite schoolboy footballers. Maturity needs further study  *British Medical Journal* | https://pubmed.ncbi.nlm.nih.gov/19351676/ | Wrong publication type |
| Broderick, C. & McKay, D.  (2009) | Reducing the risk of injury in young footballers  *British Medical Journal* | https://pubmed.ncbi.nlm.nih.gov/19297431/ | Wrong publication type |
| Materne, O. et al.  (2015) | Relationship between injuries and somatic maturation in highly trained youth soccer players  *International Research in Science and Soccer II* | N/A | Wrong publication type |
| Read, P. et al.  (2016) | The scientific foundations and associated injury risks of early soccer specialisation  *Journal of Sports Sciences* | https://pubmed.ncbi.nlm.nih.gov/27120711/ | Wrong publication type |
| Read, P. et al.  (2016) | Assessment of injury risk factors in male youth soccer players  *Strength and Conditioning Journal* | N/A | Wrong publication type |
| Sugimoto, D. et al.  (2018) | Risk factors associated with self-reported injury history in female youth soccer players  *The Physician and Sports Medicine* | https://pubmed.ncbi.nlm.nih.gov/29633890/ | Wrong study design |
| Rhodes, D. et al.  (2020) | Measures of PHV and the effect on directional dynamic stability to identify risk factors for injury in elite football  *The Journal of Sports Medicine and Physical Fitness* | https://pubmed.ncbi.nlm.nih.gov/32043345/ | Wrong study design |
| Li, X. et al.  (2023) | The associations of early specialization, sports volume, and maturity status with musculoskeletal injury in elite youth football players  *Frontiers in Physiology* | https://pubmed.ncbi.nlm.nih.gov/37250118/ | Wrong population |
| Johnson, D. et al.  (2023) | Can we reduce injury risk during the adolescent growth spurt? An iterative sequence of prevention in male academy footballers  *Annals of Human Biology* | https://pubmed.ncbi.nlm.nih.gov/37823577/ | Wrong study design |
| Monasterio, X.  (2024) | Injuries in athletic club players: growth and maturation as potential risk factors (PhD Academy Award)  *British Journal of Sports Medicine* | https://pubmed.ncbi.nlm.nih.gov/37236774/ | Wrong publication type |
| Nobari, H. et al.  (2024) | Which training load indicators are greater correlated with maturation and wellness variables in elite U14 soccer players?  *BMC Pediatrics* | https://pubmed.ncbi.nlm.nih.gov/38689258/ | Wrong study design |
